# Two Novel Hypovirulence-Associated Mycoviruses in the Phytopathogenic Fungus *Botrytis cinerea*: Molecular Characterization and Suppression of Infection Cushion Formation

**DOI:** 10.3390/v10050254

**Published:** 2018-05-13

**Authors:** Fangmin Hao, Ting Ding, Mingde Wu, Jing Zhang, Long Yang, Weidong Chen, Guoqing Li

**Affiliations:** 1 The State Key Laboratory of Agricultural Microbiology, Huazhong Agricultural University, Wuhan 430070, China; haofangmin@163.com (F.H.); dingting19910216@sina.com (T.D.); zhangjing1007@mail.hzau.edu.cn (J.Z.); yanglong@mail.hzau.edu.cn (L.Y.); guoqingli@mail.hzau.edu.cn (G.L.); 2 The Key Laboratory of Plant Pathology of Hubei Province, Huazhong Agricultural University, Wuhan 430070, China; 3 U.S. Department of Agriculture, Agricultural Research Service, Washington State University, Pullman, WA 99164, USA; w-chen@wsu.edu

**Keywords:** *Botrytis cinerea*, hypovirus, fusarivirus, hypovirulence, infection cushion

## Abstract

*Botrytis cinerea* is a necrotrophic fungus causing disease on many important agricultural crops. Two novel mycoviruses, namely Botrytis cinerea hypovirus 1 (BcHV1) and Botrytis cinerea fusarivirus 1 (BcFV1), were fully sequenced. The genome of BcHV1 is 10,214 nt long excluding a poly-A tail and possesses one large open reading frame (ORF) encoding a polyprotein possessing several conserved domains including RNA-dependent RNA polymerase (RdRp), showing homology to hypovirus-encoded polyproteins. Phylogenetic analysis indicated that BcHV1 may belong to the proposed genus Betahypovirus in the viral family *Hypoviridae*. The genome of BcFV1 is 8411 nt in length excluding the poly A tail and theoretically processes two major ORFs, namely ORF1 and ORF2. The larger ORF1 encoded polypeptide contains protein domains of an RdRp and a viral helicase, whereas the function of smaller ORF2 remains unknown. The BcFV1 was phylogenetically clustered with other fusariviruses forming an independent branch, indicating BcFV1 was a member in Fusariviridae. Both BcHV1 and BcFV1 were capable of being transmitted horizontally through hyphal anastomosis. Infection by BcHV1 alone caused attenuated virulence without affecting mycelial growth, significantly inhibited infection cushion (IC) formation, and altered expression of several IC-formation-associated genes. However, wound inoculation could fully rescue the virulence phenotype of the BcHV1 infected isolate. These results indicate the BcHV1-associated hypovirulence is caused by the viral influence on IC-formation-associated pathways.

## 1. Introduction

Fungi in the genus of *Botrytis* are able to infect more than 1400 species of cultivated plants, and are responsible for heavy losses of many important agricultural crops [1]. Among *Botrytis* spp., *B. cinerea* has the widest distribution and broadest host range, and has received most attention due to its high economic impact. The control of *B. cinerea* mostly relies on chemical fungicides [2]. However, the evolution of fungicide resistance increases the difficulty of only using fungicides for *B. cinerea* management. Therefore, some alternative methods, such as biological control, were developed for the control of *B. cinerea*. Mycoviruses, as a biocontrol agent, have been successfully used for the control of chestnut blight in Europe [3]. This consequently inspired further research of mycoviruses, and many mycoviruses were constantly reported in different groups of plant pathogenic fungi [4], although the complex vegetative compatibility groups (VCGs) limits the use of hypovirus for the control of chestnut blight in Northern America [5,6,7].

Mycoviruses are also commonly reported in the population of *Botrytis* spp., and most of these infect *B. cinerea*. Some mycoviruses infecting *Botrytis* spp. were assigned to viral families *Gammaflexiviridae*, *Alphaflexiviridae*, *Narnaviridae*, *Endornaviridae*, *Partitiviridae*, and *Totiviridae* [8,9], or the new established genus *Botybirnavirus* [10], whereas the remaining are still unassigned [8,11,12,13]. Among sequenced mycoviruses infecting *Botrytis* spp., such as Botrytis cinerea mitovirus 1 (BcMV1) [14,15], Botrytis porri botybirnavirus 1 (previously known as Botrytis porri RNA virus 1) [10], Botrytis cinerea RNA virus 1 [11] and Botrytis cinerea CCg378 virus 1 [16] were determined to be capable of attenuating virulence of *Botrytis*. Nevertheless, mycoviruses such as Botrytis ourmia-like virus [12], Botrytis cinerea negative-stranded RNA virus 1 [13], Botrytis virus F [17] and Botrytis virus X [18], seem to have no significant effects on the pathogenicity of *B. cinerea*. Recently, deep sequencing has also been employed for investigating virus diversity in *Botrytis* [19].

Hypoviruses are a group of positive single-stranded RNA (+ssRNA) mycoviruses, 9–13 kb in length excluding a poly A tail, possessing one or two open reading frames (ORFs) on their coding strands, without formation of true virions [20]. In addition to their potential for the biological control of chestnut blight, hypoviruses have also been developed as a tool for investigating interactions between viruses and host fungi [21]. Based on the phylogenetic analysis and genomic characteristics, the viral family *Hypoviridae* was proposed to be divided into two (Alphahypovirus and Betahypovirus) [22,23,24] or three genera (Alphahypovirus, Betahypovirus, and Gammahypovirus) [25].

The viral family Fusariviridae is a newly proposed +ssRNA viral family [26], probably encompassing eight viral members including Fusarium graminearum virus-DK21 (FgV-DK21) [27], Sclerotinia sclerotiorum fusarivirus 1 (SsFV1) [28], Penicillium roqueforti ssRNA mycovirus 1 (PrRV1), Rosellinia necatrix fusarivirus 1 (RnFV1) [26], Pleospora typhicola fusarivirus 1 (PtFV1) [29], Penicillium aurantiogriseum fusarivirus 1 (PaFV1) [29], Macrophomina phaseolina single-stranded RNA virus 1 (MpRV1) and Alternaria brassicicola fusarivirus 1 (AbFV1) [30]. The genomes of fusariviruses are 6–8 kb in length excluding a poly A tail, and contains two or four ORFs. The large ORF encoded polypeptides by fusariviruses usually contain an RNA dependent RNA polymerase (RdRp) domain and a viral helicase domain [30].

Although numerous mycoviruses have been reported in species of *Botrytis*, no hypovirus or fusarivirus has been documented in the population of *Botrytis*. In this study, three mycoviruses, including a hypovirus, namely Botrytis cinerea hypovirus 1 (BcHV1), a fusarivirus, namely Botrytis cinerea fusarivirus 1 (BcFV1), and an endornavirus, namely Botrytis cinerea endornavirus 1 (BcEV1), were detected in a hypovirulent *B. cinerea* strain HBtom-372. Besides these three mycoviruses, four smaller dsRNAs with the length ranging from 1.0 kb to 4.0 kb were also detected in the mycelium of strain HBtom-372. As the genome organization of BcEV1 was described previously [31], the objectives of this study were: (i) to determine the full length sequences of BcHV1, BcFV1 and four smaller dsRNAs co-infecting *B. cinerea* strain HBtom-372 in China; (ii) to investigate the biological effects of the three mycoviruses and smaller four dsRNAs on *B. cinerea*; and (iii) to elucidate the underling mechanism that may be responsible for the virus-induced hypovirulence of *B. cinerea*.

## 2. Materials and Methods

### 2.1. Fungal Strains and Culture Conditions

*B. cinerea* strains HBtom-372 and HBtom-459 were originally isolated from diseased tomato fruits in Jingmen County and Yichang County, Hubei Province, China. Strain B05.10 of *B. cinerea* (whole genome sequence available) was isolated from diseased table grape (*Vitis vinifera*) in Germany. All strains were stored as described previously [15], and working culture for each strain was established through transferring the stored mycelial plugs onto the PDA plates and subsequently incubated at 20 °C for 3 days. Isolates Z1, Z3, Z26 and Z33 were derived from strain HBtom-459 via hyphae anastomosis with HBtom-372.

### 2.2. dsRNA Extraction and Purification

Extraction and purification of dsRNA from *B. cinerea* mycelia was performed as described previously [14], the dsRNA nature was further confirmed based on resistance to DNase I and S1 nuclease (Promega, Madison, WI, USA). The extracted dsRNA was fractionated by agarose gel (1%, *w*/*v*) electrophoresis and visualized by staining with ethidium bromide (1.5 µg/L) and viewing on a UV trans-illuminator.

### 2.3. cDNA Cloning and Sequencing

The dsRNAs extracted from strain HBtom-372 of *B. cinerea* were separated by agarose gel electrophoresis. Each dsRNA band was excised and purified from the agarose gel using AxyPrep^TM^ DNA Gel Extraction Kit (Axygen Scientific, Inc.; Union City, CA, USA). The cDNA library of each dsRNA (dsRNA-A2, dsRNA-B, dsRNA-C, dsRNA-D and dsRNA-E) was produced using a random primer-mediated PCR amplification protocol [11] and sequenced as previously described [10]. The terminal sequences of each dsRNA were cloned through ligating the 3′-terminus for each strand of each dsRNA with the 5′-terminus of the 110A adaptor (Table S1) using T4 RNA ligase (Promega Corporation, 2800 Woods Hollow Road, Madison, WI, USA) at 16 °C for 18 h, and then reverse transcribed using primer RC110A (Table S1). The cDNA strands were then used as template for PCR amplification of the 5′- and 3′-terminal sequences with primer RC110A and corresponding sequence specific primer for each dsRNA segment (Figures S1 and S2, and Table S1). Cloning of the 3′- or 5′-terminal sequences of the dsRNA was performed on three separate occasions (Figures S1 and S2). The gaps between the cDNA contigs among different dsRNAs were amplified by RT-PCR with sequence specific primer pairs (Figures S1 and S2, Table S1). The dsRNA-F was agarose gel-purified, ligated with the 110A adaptor, reverse transcribed to cDNA with the primer RC110A, and the cDNA was then used as template in PCR to amplify the full length cDNA sequence of dsRNA-F directly with the primer RC110A (Figure S2) [32]. Cloning of the full-length sequence of dsRNA-F was repeated three times. All these amplicons were detected by agarose gel electrophoresis, gel-purified, and cloned into *E. coli* DH5α and sequenced as previously described [15]. All partial cDNA sequences were assembled to obtain the full-length cDNA sequence of BcHV1, BcFV1 and the other three dsRNAs (dsRNA-C, dsRNA-D, and dsRNA-E).

### 2.4. Nucleotide Sequences and Amino Acid Residues Sequences Analysis

ORFs in the full-length cDNA sequences of the dsRNAs in strain HBtom-372 of *B. cinerea* were deduced using the ORF Finder program in the website of the National Center for Biotechnology Information (NCBI, http://www.ncbi.nlm.nih.gov/gorf/gorf.html). The BlastN and BlastP programs in the public database at NCBI were used for searching the full-length cDNA sequences and deduced polypeptides of each dsRNA, respectively. CDD database (http://www.ncbi.nlm.nih.gov/Structure/cdd/wrpsb.cgi) searching deduced the domains present in the polypeptide sequence. Multiple alignment of the sequences of conserved domains in the polypeptides encoded by different mycoviruses were performed using the MUSCLE program in MEGA 5.0 [33]. Phylogenetic trees based on the sequences of conserved domains of BcHV1 and BcFV1 were constructed using the neighbor-joining (NJ) method and tested with a bootstrap of 1000 replicates to ascertain the reliability of a given branch pattern in MEGA 5.0. Putative transmembrane helices sequences were predicted using the TMHMM server version 2.0 (http://www.cbs.dtu.dk/services/TMHMM/) [34].

### 2.5. Northern Hybridization

Northern hybridization was performed to confirm the authenticity of the cDNA sequences generated from BcHV1 and BcFV1 in strain HBtom-372 of *B. cinerea*. Two DNA probes, nt positions 5514–6234 for Probe 1 and nt positions 2586–3336 for Probe 2, were designed based on full-length cDNA sequences of BcHV1 and BcFV1, respectively. The gel-purified dsRNA-A2 and dsRNA-B were separated in 1% (*w*/*v*) agarose gel and transferred to positively charged nylon membranes (Millipore, Bedford, MA, USA) [10] by the capillary transfer method using 20 × SSC as transfer buffer [35]. Probe 1 and Probe 2 were pre-labeled with the enzyme as described by the manufacturers (GE Healthcare, Little Chalfont, United Kingdom)) for hybridization with the denatured dsRNAs blotted on two membranes, respectively. The chemiluminescent signals of the probe-RNA hybrids were detected using a CDP-Star kit (GE Healthcare).

### 2.6. Biological Properties of Botrytis cinerea Strain HBtom-372

MAPs (mycelium agar plugs, 6 mm in diameter) removed from the colony margin of a 2–4-day-old culture of each strain or isolate were placed on PDA in petri dishes (9 cm in diameter), one plug per dish. The dishes were incubated at 20 °C for determination of the mycelial growth rate and for observation of the colony morphology. Lesion diameter on rapeseed (*Brassica napus* L.) (20 °C, 72 h) and tomato (*Lycopersicon esculentum* Mill.) leaves (20 °C, 48 h), and radial mycelial growth rate on PDA (20 °C, in the dark) was determined using the procedures described in our previous studies [14,15].

To rule out the possibility that the derivative isolates were contaminated by the donor strain, the genetic backgrounds of all the derived isolates, along with strains HBtom-372 and HBtom-459, were profiled by randomly amplified polymorphic DNA (RAPD) using the 10-mer primer OPC-04 as described previously [36] (Table S1).

### 2.7. Viral Horizontal Transmission and Detection of Mycoviruses by RT-PCR

Horizontal transmission refers to the transmission of hypovirulence-associated dsRNAs from hypovirulent to virulent fungal strains through hyphal anastomosis or contact [37]. The experiment was carried out by using the pairing culture technique as previously described [14,15]. In each pairing culture (9 cm in diameter), the dsRNA-harboring hypovirulent strain HBtom-372 served as the donor, whereas strains HBtom-459 served as the recipient. Derivative isolates were obtained from the recipient strain HBtom-459 in the contact cultures using the method described by Wu et al. [14]. All derivative isolates were subjected to test for the presence of three mycoviruses and other dsRNAs through RT-PCR with primer pairs H-RT-F/H-RT-R, F-RT-F/F-RT-R, C-RT-F/C-RT-R, and RT-F-F/RT-F-R, respectively (Table S1). Strains HBtom-372 and HBtom-459 were included as controls in this experiment. Four derivative isolates, namely Z1, Z3, Z26 and Z33, were selected and individually tested for the pathogenicity on both intact and wounded detached rapeseed leaves (20 °C, 72 h), mycelial growth rate on PDA (20 °C), production of conidia and sclerotia, and the presence of dsRNA in mycelia. In addition, 98 strains of *B. cinerea* from different places of China (Table S2) were subjected to test for the presence of BcHV1 and BcFV1.

### 2.8. Stereomicroscopic Observation of Infection Cushions

The MAPs (6 mm diameter) of strains HBtom-372 and HBtom-459 and their derivative isolates of *B. cinerea* were inoculated on onion bulb scales, one MAP per each bulb scale, three scales for each isolate/strain. All inoculated onion scales were placed on moistened paper towels in plastic trays and covered with transparent plastic films to maintain high humidity. After incubation at 20 °C for 9 h, MAPs and the bulb scales of onion were stained with methyl blue and examined for formation of ICs under a stereomicroscope. The number of ICs formed by each *B. cinerea* isolate/strain around the MAPs was counted.

### 2.9. Quantitative Real-Time PCR

The mycelia of strain HBtom-459 and isolate Z33 were harvested from PDA plate and onion bulb scales (9 h post inoculation), respectively, and the total RNA was extracted from the harvested mycelia with TRIzol^®^ reagent (Invitrogen Corp, Carlsbad, CA, USA) using the procedures recommended by the manufacturer. The extracted RNA was then used for detection the expression of IC formation-associated genes by RT-PCR and qRT-PCR with the primer sets listed in Table S3. The calculation of the relative expression level of each gene was done using the procedures described previously [38]. The experiment was repeated two more times. 

## 3. Results

### 3.1. Botrytis cinerea Strain HBtom-372 Exhibits Hypovirulence Traits

**Figure 1:**
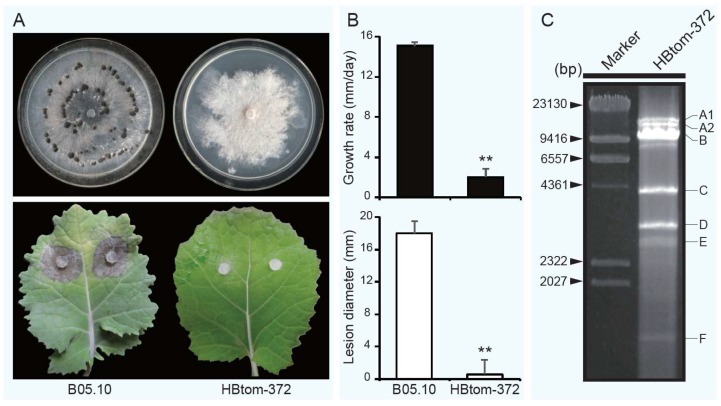
Biological properties and dsRNA detection of *Botrytis cinerea* strain HBtom-372 and B05.10. (**A**) Colony morphology (upper, 20 °C, 15 days) and pathogenicity assay (lower, 20 °C, 3 days) of strains HBtom-372 and B05.10 on potato dextrose agar (PDA) and detached rapeseed leaves, respectively. (**B**) Radial mycelial growth rate (20 °C, upper) on PDA and lesion diameter (20 °C, 72 h, lower) on detached rapeseed leaves of strains HBtom-372 and B05.10. “**” indicates a significant difference (*p* < 0.01) between strains HBtom-372 and B05.10 in both pathogenicity and radial mycelial growth rate. (**C**) Agarose gel electrophoresis of dsRNAs extracted from the mycelium of *Botrytis cinerea* strain HBtom-372. Marker, λ-*Hin*d III digest DNA marker.

After cultivation on potato dextrose agar (PDA) for 15 days, strain HBtom-372 formed abnormal colonies with no production of conidia and sclerotia, and was unable to cover the entire Petri dishes (Figure 1A). In contrast, strain B05.10 formed normal colonies with the formation of conidia and sclerotia (Figure 1A). It is notable that strain HBtom-372 also failed to produce conidia and sclerotia on PDA even after 30 days. The virulence assay on detached rapeseed leaves revealed that the average lesion diameter (0.5 mm) caused by strain HBtom-372 was significantly smaller than that (18.0 mm) of strain B05.10 (Figure 1A,B, Table S6). Compared with the virus free strain B05.10, the radial mycelial growth of HBtom-372 on PDA was significantly slower. The average radial mycelial growth rate of strain HBtom-372 was 2.0 mm/day, which was significantly slower than that of strain B05.10 (15.1 mm/day). Virulence assay on detached tomato leaves also indicated that the virulence of strain HBtom-372 was significantly reduced compared with that of strain HBtom-459 (Figure S3). After DNase I and S1 nuclease digestion, multiple dsRNA segments were detected through electrophoresis in the mycelium of HBtom-372 of *B. cinerea* with the sizes ranging from 13.5 kb to 1.5 kb, named from largest to smallest as dsRNA-A1, dsRNA-A2, dsRNA-B, dsRNA-C, dsRNA-D, dsRNA-E and dsRNA-F (Figure 1C), whereas no dsRNA was detected in HBtom-459 (Figure S3). A faint dsRNA segment close to dsRNA-B was also observed. However, no other sequences except BcFV1 (dsRNA-B) were obtained through the sequencing of the cDNA library (about 60 clones) constructed by the gel-purified dsRNA. Therefore, we suppose this band may also be derived from dsRNA-B. Among these dsRNAs, dsRNA-A1 was previously determined to be BcEV1. It is notable that the dsRNA of BcEV1 was purified from the agarose gel and then detected through electrophoresis once more, thus only one dsRNA segment was shown in our previous study [31].

### 3.2. Full-Length cDNA Sequences of BcHV1 and BcFV1

**Figure 2:**
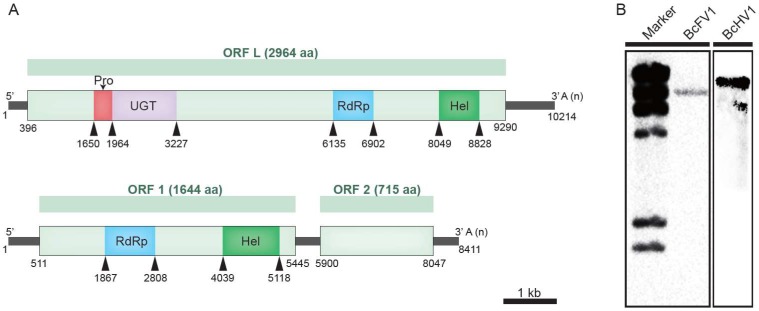
(**A**) Schematic diagram of the genome organization of Botrytis cinerea hypovirus 1 (BcHV1) and Botrytis cinerea fusarivirus 1 (BcFV1). The coding strand of BcHV1 is 10,214 nt long and contains a large ORF encoding a polyprotein of 2964 aa, possessing conserved domains: Prot, papain-like protease; UGT, UDP glucose/sterol glucosyltransferase; RdRp, RNA-dependent RNA polymerase; Hel, viral helicase superfamily. The coding strand of BcFV1 is 8411 nt in length and contains two major ORFs, ORF1 and ORF2, encoding two polypeptides of 1644 aa and 715aa, respectively. ORF1-encoded polypeptide possesses two conserved domains, RdRp and Hel. (**B**) Northern blotting detection of BcHV1 and BcFV1 dsRNAs extracted from the mycelium of *B. cinerea* strain HBtom-372. Marker, λ-Hind III digest DNA marker.

The assembled full length genome sequence of BcHV1 (dsRNA-A2) is 10,214 nt, with a GC content of 44.7%, excluding the poly A tail (GenBank accession No. MG554632). The genome of BcHV1 was hypothesized to contain one large ORF with two 395 nts and 924 nts long untranslated regions (UTR) located at both 5′- and 3′- terminus of the positive strand of BcHV1, respectively. The large ORF, namely ORF L, was predicted to encode a putative polypeptide of 2964 amino acid (aa) residues with a deduced molecular mass of 336 kDa (Figure 2A). The BLASTp analysis of the polypeptide showed that ORF L-encoded polypeptide is closely related to Sclerotinia sclerotiorum hypovirus 1 (SsHV1, 67.4% identity), Cryphonectria hypovirus 3 (CHV3, 58.31% identity), Phomopsis longicolla hypovirus 1 (PlHV1, 57.84% identity) and Valsa ceratosperma hypovirus 1 (VcHV1, 56.48% identity) (Table S4). Therefore, we supposed that BcHV1 should be a member in the viral family *Hypoviridae*.

The full length cDNA sequence of BcFV1 (dsRNA-B) was 8411 bp long with a GC content of 46.4%, excluding the poly A tail (GenBank accession No. MG554633). The whole genome of BcFV1 possesses two large ORFs and two 510-nt and 364-nt long untranslated regions (UTR) located at both 5′- and 3′- terminus of the positive strand of BcFV1, respectively (Figure 2A). A 456 nts long UTR was located between the two ORFs. The ORF 1 and ORF 2 were predicted to encode a 186 kDa and an 81.7 kDa putative polypeptide of 1644 and 734 aa residues in length, respectively. The BLASTp analysis of ORF 1-encoded polypeptide of BcFV1 showed that it was related to polypeptides encoded by AbFV1 (25.58% identity), SsFV1 (24.3% identity) and PtFV1 (23.84% identity). In addition, the polypeptide encoded by ORF 1 of BcFV1 also showed low sequence similarity to viruses of *Hypoviridae*, *Potyviridae*, *Poxviridae*, *Iflaviridae* and *Secoviridae* (Table S5). However, the polypeptide encoded by ORF 2 showed no significant sequence similarity with proteins in the database of NCBI by using BlASTp search. Therefore, we suppose that BcFV1 might be a novel member in the proposed virial family Fusariviridae. Moreover, the viral sequences of both BcHV1 and BcFV1 were confirmed through the Northern hybridization analysis (Figure 2B). Considering both viruses possess +ssRNA genomes, the observed dsRNAs are likely intermediates during viral replication.

### 3.3. Putative Polyprotein Encoded by BcHV1 and BcFV1

**Figure 3:**
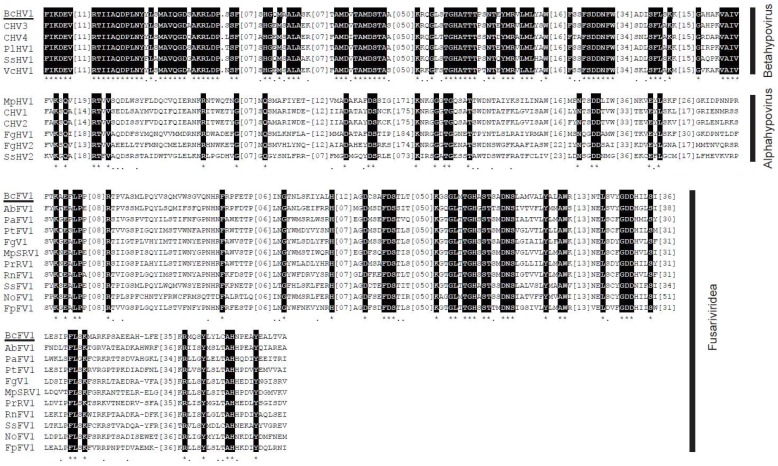
Multiple alignment of the amino acid sequences of RNA-dependent RNA polymerase domains in the polyprotein encoded by Botrytis cinerea hypovirus 1 (BcHV1) and Botrytis cinerea fusarivirus 1 (BcFV1), respectively. “*” indicates identical amino acid residues; and “.” indicate low chemically similar amino acid residues. The abbreviations of virus names are listed in Tables S4 and S5. The names of BcHV1 and BcFV1 are underlined.

CDD database search of the BcHV1-encoded polypeptide in the database of NCBI revealed that it contained a putative papain-like protease (Prot) domain, a UDP glucose/sterol glucosyltransferase (UGT) domain, an RdRp domain and a viral RNA Helicase (Hel) domain (Figure 2A). The predicted RdRp domain was located between the UGT domain and Hel domain, including eight conserved motifs (I–VIII) (Figure 3) as described in other hypoviruses [39]. The RdRp domain of BcHV1 was closely related to SsHV1 (85.94% identity) and PlHV1 (85.16% identity) (Table S4). A typical Prot domain with conserved predicted autoproteolytic catalytic site (at positions Cys^426^ and His^473^) and a putative polyprotein cleavage site (at position Gly^523^) was detected in the polyprotein encoded by BcHV1 based on sequence alignment with other hypoviruses (Figure 2A, Table S4, and Figure S4). CDD database search showed that BcHV1 also contained a conserved UGT domain as reported in SsHV1 and other hypoviruses [40] (Figure 2A and Figure S4). Three characteristic motifs, namely GKST box, DExH box and QRxGR box, of the predicted Hel domain of BcHV1 were also detected through multiple sequence alignment (Figure S4).

The BcFV1 ORF 1-encoded polypeptide contained a putative RdRp domain and a viral Hel domain (Figure 2A). The predicted RdRp domain included eight conserved motifs (I–VIII) and was closely related to RdRp domains of other fusariviruses, especially AbFV1 (47.17% identity) and SsFV1 (46.98% identity) (Table S5 and Figure 3). Six conserved motifs in the viral Hel of fusariviruses were also detected in the Hel of BcFV1 through multiple sequence alignment (Table S5 and Figure S4). However, no protein showed significant similarity to the ORF 2 encoded protein through BLASTp search in NCBI database. Similar to other fusariviruses, transmembrane (TM) domains (Figure S5) were also found at the *N*-proximal ORF 1-coded protein of BcFV1 [34].

### 3.4. Phylogenetic Analysis of BcHV1 and BcFV1

**Figure 4:**
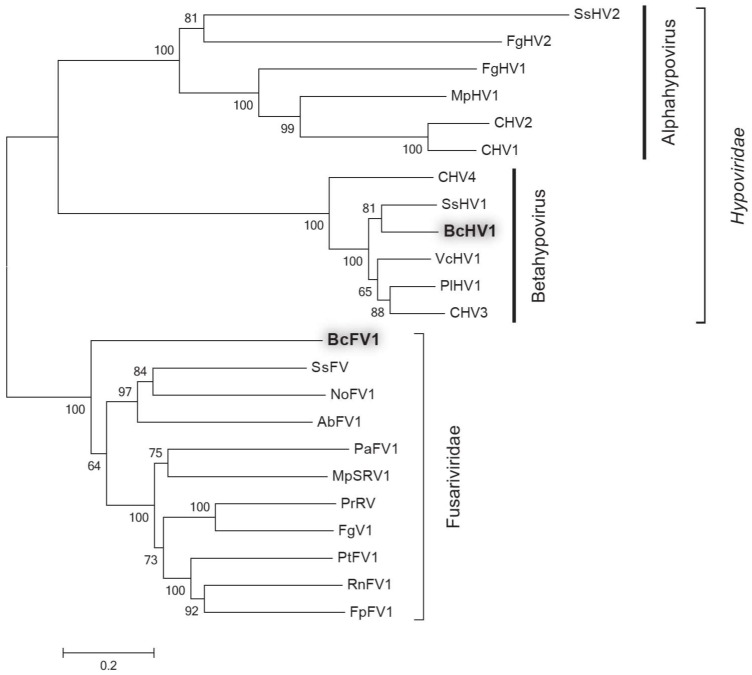
Phylogenetic analysis of Botrytis cinerea hypovirus 1 (BcHV1) and Botrytis cinerea fusarivirus 1 (BcFV1) based on RdRp-Hel region from strain HBtom-372 of *B. cinerea*. The abbreviations of virus names for constructing the phylogenetic tree are listed in Tables S4 and S5.

To define the phylogenetic relationship of BcHV1 and BcFV1 with other mycoviruses (Table S4), a phylogenetic tree was established based on the RdRp-Hel region including the RdRp domain, Hel domain and the aa sequence between the two domains. Three major clades, namely Alphahypovirus, Betahypovirus, and Fusariviridae, were observed in the RdRp-Hel phylogenetic tree with bootstrap of 100% for each clade (Figure 4). BcHV1 appeared to be mostly close related to SsHV1 with bootstrap of 81%, and then clustered with other betahypoviruses forming an independent Betahypovirus clade. Although BcFV1 was mostly homologous to AbFV1 through BLAST search, BcFV1 did not cluster with AbFV1, instead of forming an independent branch, and then clustered with other fusariviruses forming the Fusariviridae clade (Figure 4).

### 3.5. Nucleotide Sequence of other dsRNAs

**Figure 5:**
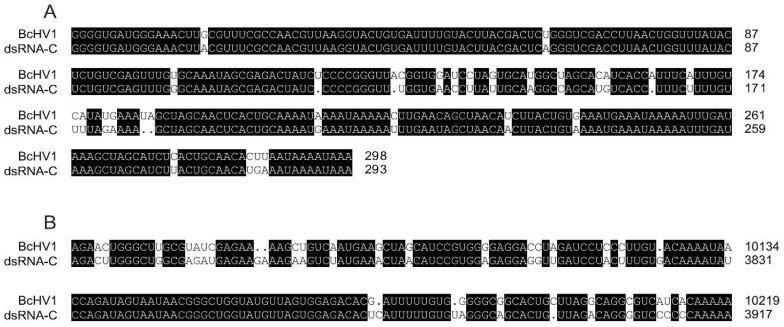
Alignment of the 5′ (**A**) and 3′-terminal (**B**) sequences of the coding strands of Botrytis cinerea hypovirus 1 (BcHV1) and dsRNA-C. An identical nucleotide is highlighted with black shading. The black dot indicates a missing nucleotide.

The full-length cDNA sequences of remaining four dsRNAs, namely dsRNA-C, dsRNA-D, dsRNA-E and dsRNA-F, were also determined. Sequence analysis indicated that dsRNA-C (GenBank accession No. MG554634) and dsRNA-F (GenBank accession No. MG554637) were 3912 bp and 1375 bp in length excluding the Poly A tail, respectively. Alignment of the cDNA sequences of dsRNA-C and dsRNA-F showed that the nucleotide (nt) sequence of dsRNA-F (1-1375) was 100%, 99.57% and 98.91% identical to three regions of dsRNA-C, namely 1–171, 2451–3142 and 3401–3912, respectively (Figure S6). Thus, dsRNA-F might be the defective RNA of dsRNA-C. Both the 5′- and 3′-terminal sequences of dsRNA-C were closely related to those of BcHV1. Sequence alignment showed that of the first 298 nts sequences at 5′-termini of SsHV1 and dsRNA-C were 88.93% identical (Figure 5), and their last 202 nts at the 3′-termini were 83.65% identical (Figure 5). However, no significant sequence similarity was detected in the middle region between dsRNA-C and BcHV1 at nt level. DsRNA-C was deduced to encode a protein of 670 aa with an approximate molecular mass of 73.8 kDa. However, no homologous protein and conserved domain were detected through BLASTp and CDD search with the deduced aa sequence of dsRNA-C.

Sequence analysis indicated that the full length cDNA of dsRNA-D (GenBank accession No. MG554635) and dsRNA-E (GenBank accession No. MG554636) were 3482 bp and 3253 bp in length, respectively. The nt sequences of dsRNA-D and dsRNA-E were almost 100% identical to two regions, namely nt 1–3480 and 1–3247, of BcFV1, respectively, except few nt insertions at both 5′-termini and middle region of the two dsRNAs (Figure S6). Therefore, we suppose that dsRNA-D and dsRNA-E may be two defective RNAs of BcFV1.

### 3.6. Horizontal Transmission of Hypovirulence-Associated dsRNAs

**Figure 6:**
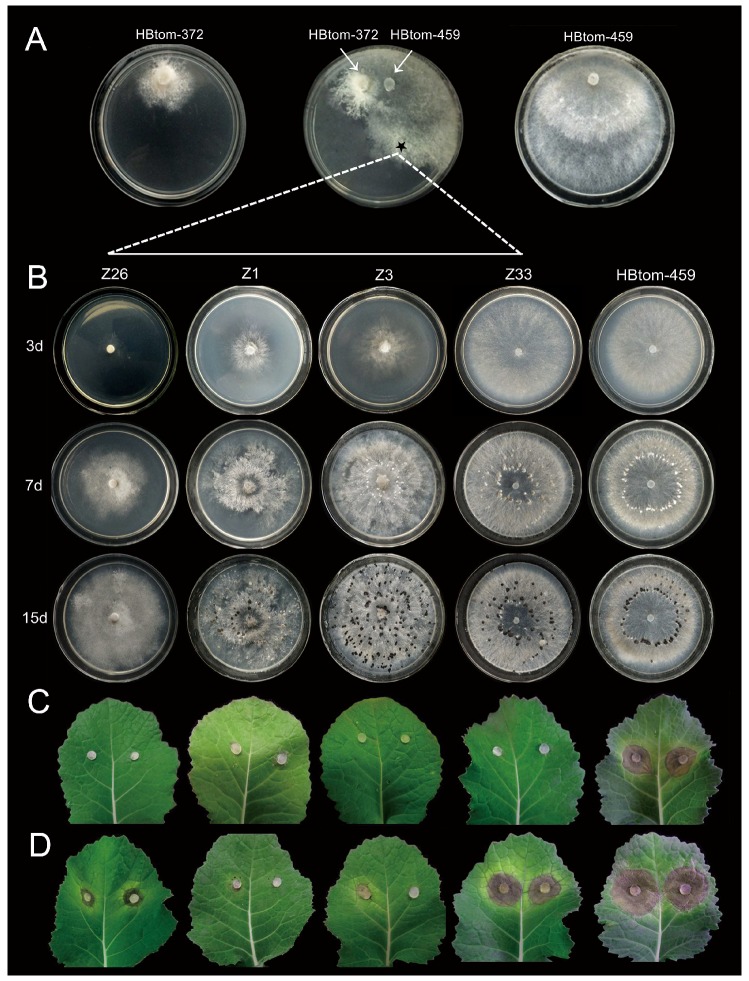
Horizontal transmission of three mycoviruses and biological properties of derivative *Botrytis cinerea* isolates. (**A**) Colony morphology of virus-infected strain HBtom-372, virus-free strain HBtom-459 and dual culture of these two strains allowing horizontal transmission of viruses from strain HBtom-372 to strain HBtom-459 through hyphal anastomosis on potato dextrose agar (PDA) (20 °C, seven days). (**B**) Colony morphology among strain HBtom-459 and derivative *B. cinerea* isolates and on PDA at 20 °C for 3, 7 and 15 days, respectively. Pathogenicity assay on intact (**C**) and wounded (**D**) detached rapeseed leaves (*Brassica napus*) of derivative *B. cinerea* isolates and strain HBtom-459. The star in the dual culture indicates the area where mycelial agar plugs were taken for generation of derivative isolates.

**Figure 7:**
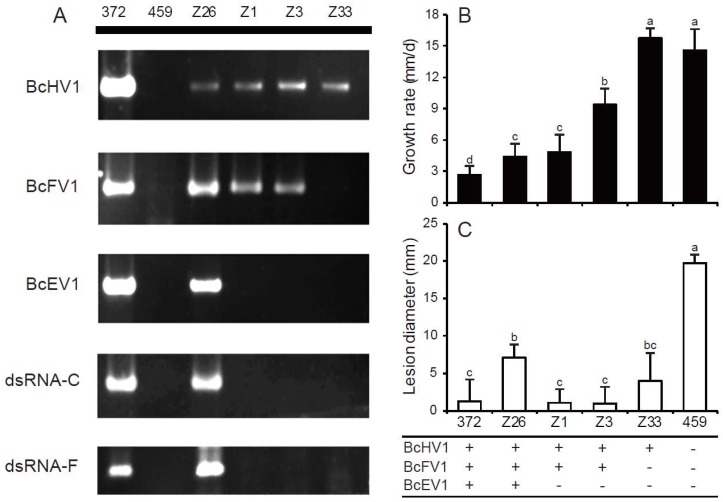
The radial mycelial growth and virulence of *B. cinerea* isolates/strains infected with different mycoviruses. (**A**) The reverse transcription (RT)-PCR detection of Botrytis cinerea endornavirus 1 (BcEV1), Botrytis cinerea hypovirus 1(BcHV1), Botrytis cinerea fusarivirus 1 (BcFV1), dsRNA-C and dsRNA-F in *B. cinerea* strains with primer pairs listed in Table S1. The radial growth rate on potato dextrose agar at 20 °C (**B**) and lesion diameter on detached leaves of rapeseed leaves (20 °C, 72 h) (*Brassica napus*) (**C**) for *B. cinerea* strains/isolates. The names of strains HBtom-372 and HBtom-459 are abbreviated here as 372 and 459, respectively. Bars represent arithmetic mean ± S.E.M. In each histogram, bars labeled with the same letters are not significantly different (*p* > 0.05) according to the least-significant-difference test. The symbols “+” and “−” indicate the presence and absence of corresponding virus, respectively.

To determine the transmission capacity of BcHV1, BcFV1 and BcEV1, strain HBtom-372 was dually cultured with strain HBtom-459 on the same PDA plate, while the inhibition of mycelial growth on the margin of HBtom-459 was observed after seven days (Figure 6A and Figure S7). Several mycelial plugs were picked out from the abnormal margin for establishing the derivative isolates, and 33 isolates were obtained. The presences of three mycoviruses, dsRNA-C and dsRNA-F in all 33 derivative isolates were detected through reverse transcription (RT)-PCR with the specific primers (Table S1). The results showed that isolate Z33 only contained one virus, BcHV1, and isolate Z26 contained all three viruses, BcEV1, BcHV1 and BcFV1, while the other isolates including Z1 and Z3 were infected by both BcHV1 and BcFV1 (Figure 7A). The dsRNA-C and dsRNA-F were only detected in isolate Z26, while not detected in isolates Z33, Z1 and Z3 (Figure 7A). All four derived isolates showed same RAPD profiles as those of their recipient HBtom-459, indicating that the derived isolates were not from contamination (Figure S8). Four isolates, Z1, Z3, Z26 and Z33, were selected for further biological characterization.

Isolate Z33 grew fast on PDA plate with the average radial growth rate of 15.8 mm/day, and was comparable to strain HBtom-459 with the radial growth rate of 14.6 mm/day (Figure 7B and Table S6). The other strain or isolates grew slower than both isolate Z33 and strain HBtom-459 with the average radial growth rate ranging from 2.6 mm/day to 9.4 mm/day (Figure 7B and Table S6). Although both isolates Z1 and Z3 were infected by BcFV1 and BcHV1, isolate Z3 grew slightly faster than isolate Z1. The culture morphology of isolate Z33 was similar to that of strain HBtom-459 without the formation of mycelial sectors, and both strains colonized the entire plate within three days (Figure 6B). However, isolates Z1 and Z3 were unable to colonize the entire plate within three days and formed many mycelial sectors after 5 days (Figure 6B). Isolate Z26 was severely debilitated with the formation of many mycelial sectors, and could not colonize the entire plate within seven days (Figure 6B). The production of conidia and sclerotia varied in different derivative strains (Table 1). Compared with strain HBtom-459, the yields of conidia were slightly decreased in isolate Z33, Z1 and Z3, whereas the yields of sclerotia were significantly increased in isolate Z33, Z1 and Z3, with 79, 66 and 115 sclerotia per dish, respectively, but with a smaller size (Table 1). However, similar to strain HBtom-372, isolate Z26 formed no conidia and sclerotia after 30 days (Figure 6B).

The virulence assay on intact detached rapeseed leaves showed that the virulence of all derivative isolates were significantly impaired (Figure 6C). The average lesion diameters on rapeseed leaves were 4.7 mm, 0.4 mm, 0.0 mm, and 3.8 mm for isolates Z26, Z1, Z3, and Z33, respectively, which were apparently reduced in comparison with strain HBtom-459 of the average lesion diameter of 16.5 mm (Figure 6C and Figure 7C, and Table S6).

### 3.7. Formation of Infection-Cushions

**Figure 8:**
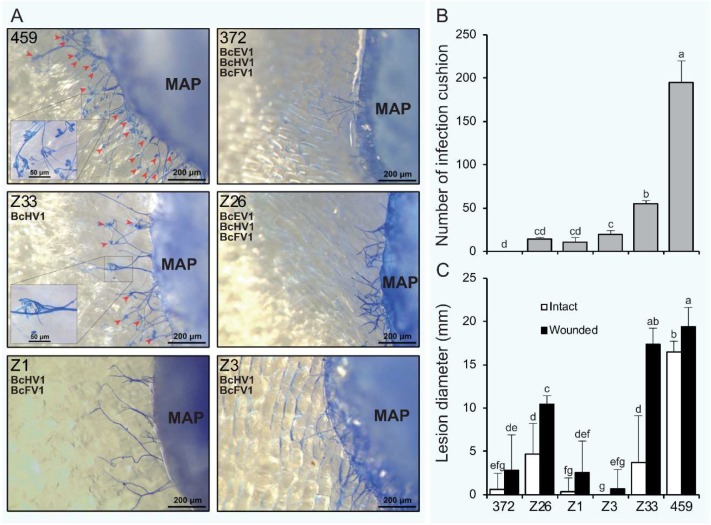
Infection cushion formation (IC) and virulence of *Botrytis cinerea* virus-infected strain HBtom-372 (372), virus-free strain HBtom-459 (459) and virus-infected isolates Z1, Z3, Z26 and Z33 derived from HBtom-459; (**A**) Comparison of IC (red arrowheads) formation on epidermis of onion bulbs after staining with methyl blue. The mycoviruses harbored in each isolate/strain are indicated. The MAP = mycelial agar plug. (**B**) Number of ICs formed around the mycelial agar plugs (6 mm diam). (**C**) Lesion diameters formed on intact and wounded detached rapeseed leaves (*Brassica napus*). (**B**,**C**) Bars represent arithmetic mean ± S.E.M. In each histogram, bars labeled with the same letters are not significantly different (*p* > 0.05) according to the least-significant-difference test.

After inoculation on onion bulb epidermis for 9 h, lots of infection cushions (ICs) were formed around the mycelial agar plugs (MAPs) of strain HBtom-459 with the average number of 195 (Figure 8A,B, Figure S9, and Table S6). In contrast, the numbers of ICs formed by four derivative isolates were dramatically decreased in varying degrees, and the average numbers of ICs formed by isolates Z1, Z3, Z26 and Z33, were 11, 20, 15, and 55, respectively (Figure 8B and Table S6). However, no IC was observed around the MAPs of strain HBtom-372. To test the association of hypovirulence to the decreased formation of ICs, the MAPs of each strain/isolate were inoculated on both intact and wounded rapeseed leaves. The results showed that the virulence on wounded rapeseed leaves was significantly enhanced compared with that on intact rapeseed leaves for most *B. cinerea* strains/isolates (Figure 6D and Figure 8C). It is notable that the virulence of isolate Z33 was almost fully recovered on wounded leaves, as the average lesion diameter on wounded leaves (17.4 mm) was dramatically increased in comparison with that on intact leaves (3.8 mm) (Figure 8C and Table S6). More interestingly, the virulence of isolate Z33 on wounded leaves was also comparable to that of strain HBtom-459 on either intact leaves (16.5 mm) or wounded leaves (19.4 mm) (Figure 6D and Figure 8C, and Table S6).

### 3.8. Transcripts of Infection Cushion Formation-Associated Genes

The functions of six infection cushion formation associated genes are summarized in Table 2. Among the six genes, three genes, *Bciqg1* [41], *Bcpdi1* [42] and *Bcmsb2* [43], were previously characterized in *B. cinerea*, whereas the other three genes are homologs of IC-associated genes in the closely related species *S. sclerotiorum* [44,45,46]. Bciqg1 is a scaffold that mediates interaction of the catalytic subunits with the regulator BcNoxR, which is involved in the MAP kinase- and calcium-dependent signaling pathways [41]. The Bcpdi1 is the essential endoplasmic reticulum (ER) protein as an interaction partner of the NoxA complex, and affects the redox homeostasis in *B. cinerea* [42]. The function of Bcmsb2 is likely to sense hard surfaces for germ tubes and hyphae that triggers the formation of appressoria or ICs via the activation of the BMP1 MAP kinase pathway [43]. The Bcsac1 is the homolog of *S. sclerotiorum* adenylate cyclase (sac1), probably participating in the cAMP-signaling pathway in *B. cinerea*. The homolog of Bcrgb1 in *S. sclerotiorum* (rgb1) was determined to be the regulatory B subunit of the Type 2A phosphoprotein phosphatase (PP2A) involved in several cellular signal-transduction pathways [46]. Sscaf1, the homolog of *B. cinerea* Bccaf1, is a secretory protein and possesses a putative Ca^2+^-binding EF-hand motif [44].

**Figure 9:**
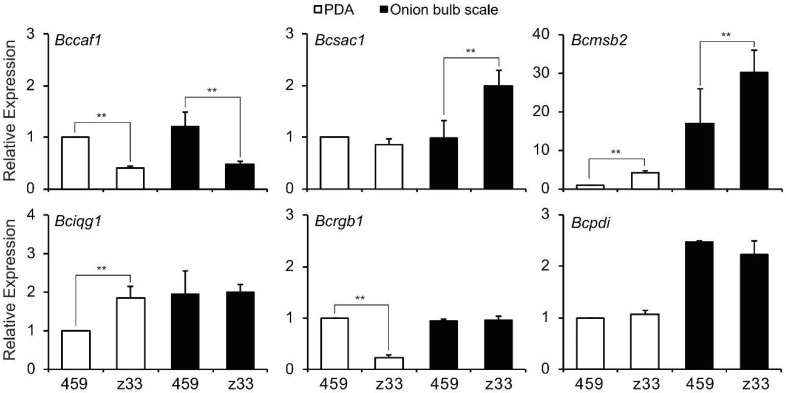
Comparison of relative transcript levels of six infection cushion formation-associated genes including *Bccaf1*, *Bcsac1*, *Bcmsb2*, *Bciqg1*, *Bcrgb1*, and *Bcpdi1* in strain HBtom-459 (459) and isolate Z33 of *B. cinerea* on PDA or onion bulb scale. The results in each histogram are expressed as arithmetic means with the standard errors of the means. “**” indicates a significant difference (*p* < 0.01) between strain HBtom-459 and isolate Z33 in relative transcript level according to the Student *t* test.

The transcriptions of six IC formation-associated genes were investigated using RT-PCR and quantitative real-time PCR (qRT-PCR) (Figure 9). Compared with strain HBtom-459, only one gene, *Bccaf1*, showed a decreased transcription in isolate Z33 on onion bulb scale, while the transcriptions of two genes, *Bcmsb2* and *Bcsac1*, were increased. Three genes including *Bciqg1*, *Bcpdi1* and *Bcrgb1* transcribed in isolate Z33 at almost same levels as in strain HBtom-459 on onion bulb scale. On PDA plate, the transcriptions of genes *Bccaf1* and *Bcrgb1* were down-regulated, whereas transcriptions of genes *Bcmsb2* and *Bciqg1* were up-regulated in isolate Z33 compared with strain HBtom-459.

### 3.9. Incidence and Distribution of BcHV1 and BcFV1

Both BcHV1 and BcFV1 were widely distributed in populations of *B. cinerea*, as about 29.6% and 14.3% of *B. cinerea* strains carried BcHV1 and BcFV1 as detected by RT-PCR, respectively (Table S2). It is notable that 13 BcFV1-infected *B. cinerea* strains were co-infected with BcHV1, while only one strain of *B. cinerea* was infected by BcFV1 without BcHV1. The geographic distributions of the two viruses were vast, of which BcHV1 was detected in all five provinces, while BcFV1 was detected in three of five provinces.

## 4. Discussion

In the present study, we described the molecular and biological properties of two mycoviruses, BcHV1 and BcFV1, co-infecting the hypovirulent strain HBtom-372 of *B. cinerea* with BcEV1. Notwithstanding numerous mycoviruses have been reported in *Botrytis* spp., no hypovirus or fusarivirus has been described in species of *Botrytis* [8].

Sequence and phylogenetic analyses of the putative polyprotein encoded by BcHV1 strongly suggested that BcHV1 belonged to the viral family *Hypoviridae.* Phylogenetic tree based on the RdRp-Hel region clearly supported that the viral family *Hypoviridae* could be divided into two proposed genera Alphahypovirus and Betahypovirus, wherein BcHV1 was clustered with SsHV1, CHV3, CHV4, PlHV1 and VcHV1 forming an independent Betahypovirus clade (bootstrap support 100%). Excluding BcHV1, pairwise sequence analysis of the rest hypoviruses in the Betahypovirus clade showed that they shared 49.9–64.4% nt and 47.6–68.6% aa sequence identity with each other. The pairwise identities of BcHV1 with the other five betahypoviruses range from 49.5% to 65.8% and from 46.8 to 67.4% at levels of the nt sequence and aa sequence, respectively. Considering the virus host, phylogenetic analysis, and sequence identity with other hypoviruses, BcHV1 might be a novel hypovirus isolated from *B. cinerea.* Although BcFV1 clustered with other fusariviruses through phylogenetic analysis, BcFV1 only showed relatively low sequence similarity with those fusariviruses (Table S5). Thus, BcFV1 should be considered as a novel viral member in the proposed viral family Fusariviridae isolated from *B. cinerea.* BLAST analysis also indicated that BcFV1 (nt region 1807–2822) showed 70.96% nt and 83.08% aa identity with those of a virus-like contig (grapevine associated mycovirus-2, GaMV2, GU108600) associated with grapevine plants [47]. Therefore, GaMV2 might be a strain or relative of BcFV1. As *B. cinerea* is a common fungal pathogen of grape, GaMV2 may also be carried by *B. cinerea* isolates infecting grape.

Cryphonectria hypovirus 1 (CHV1), the type species of the genus *Hypovirus*, has been successfully used for the control of chestnut blight caused by *C. parasitica* [40] in Europe. Although the complex VCGs limiting the use of hypovirus for the control of *C. parasitica* [5,6,7], successful engineering super mycovirus donor strains makes the control of *C. parasitica* with hypovirus in Northern America more hopefully in future [48]. Not only used for biocontrol, the CHV1-*C. parasitica* system has also be used as tools for deciphering molecular mechanisms involved in interaction between viruses and hosts [21]. In addition to *B. cinerea*, hypoviruses have been reported in eight other plant pathogenic fungal species, including *C. parasitica* [49], *S. sclerotiorum* [40], *Fusarium graminearum* [24], *F. poae* [50], *F. langsethiae* [51], *Valsa ceratosperma* [22], *Macrophomina phaseolina* [52] and *Phomopsis longicolla* [53]. The effects of hypovirus infection varied in different virus–host systems. The infections of FgHV1, CHV4/SR2 and VcHV1/MVC86 have no significant effect on their hosts [22,54,55], whereas CHV1/EP713, CHV2/NB58, CHV3/GH2 and FgHV2 infections caused or were associated with the hypovirulence of their hosts [24,39,56,57,58,59]. In *S. sclerotiorum*, it was shown that co-infection of SsHV1/SZ150 and its satellite-like small dsRNA, not SsHV1/SZ150 alone, caused the hypovirulence [40]. As only 5′- and 3′- terminal sequences of dsRNA-C were homologous to those of BcHV1 and the hypothetical protein of dsRNA-C possessed no RdRp domain, dsRNA-C was believed to be unable to replicate independently and should be the satellite RNA of BcHV1. However, the infection of BcHV1 alone without the satellite RNA dsRNA-C (in strain Z33) significantly reduced virulence without affecting mycelial growth (Figure 7B, Table S6). Moreover, isolates Z1, Z3 and Z33 of *B. cinerea* lacking the satellite RNAs (dsRNA-C and dsRNA-F) also showed the hypovirulence trait. Therefore, the presence of BcHV1, not the satellite RNAs, is associated with the hypovirulence of strain HBtom-372.

Among seven reported fusariviruses, SsFV1, RnFV1, AbFV1, PtFV1, PaFV1, FpFV1 and FgV1, only FgV1 showed a significant association with pronounced morphological changes, like impaired mycelial growth, increased pigmentation, reduced mycotoxin production and attenuated virulence of its host [60]. The other fusariviruses, like PtFV1, FpFV1 and PaFV1, lack the information of their biological properties [29,50]. SsFV1 and RnFV1 were able to be horizontally transmitted via anastomosis, but their infection did not cause any symptoms [26,28]. In addition, AbFV1 was co-infected with Alternaria brassicicola endornavirus 1 in *Alternaria brassicicola* strain 817-14. As AbFV1-cured strain showed comparable biological properties with AbFV1-infected strain, AbFV1 was believed to have no conspicuous impact on its host [30]. In the present study, we obtained a series derivative isolates of *B. cinerea*, unfortunately, no derivative isolate was infected by BcFV1 alone. Compared with isolate Z33 (only carrying BcHV1), the derivative isolates Z1 and Z3 (carrying both BcHV1 and BcFV1) showed more severe debilitation. Isolate Z33 only showed impaired virulence, whereas isolates Z1 and Z3 also showed slower mycelial growth and abnormal colony morphology besides impaired virulence (Figure 6). Moreover, the derivative isolate Z26 (carrying BcEV1, BcHV1 and BcFV1), showed almost the same phenotype of the donor strain HBtom-372 (Figure 6). Thus, the co-infection of BcHV1 with BcFV1, or with both BcEV1 and BcFV1, may exacerbate the debilitation of virus-infected strain at varying degrees. Since no isolate solely infected by BcFV1 or BcEV1 was obtained, the effect of BcFV1 on *B. cinerea* biology remains to be investigated in the future. In addition, most derivative isolates were co-infected with BcHV1 and BcFV1 or infected by BcHV1 alone, indicating the faster transmission of BcHV1 and BcFV1 in comparison with BcEV1 during hyphal anastomosis. In addition, only BcHV1 solely infected isolate was detected, also suggesting BcHV1 might be transmitted faster than BcFV1 through hyphal anastomosis.

Although hypovirulence-associated mycoviruses were recorded in many phytopathogenic fungi, the underling mechanism responsible for virus-mediated hypovirulence remains unclear. The impact of CHV1/EP713 on *C. parasitica* seems to be global, besides hypovirulence, multiple phenotypical changes including colony pigmentation, ascospore production and female sterility were also observed [37], accompanying with the transcriptional alternation of hundreds of genes involved in over a dozen pathways [61,62]. In addition, comparative proteomics analysis also indicated dozens of vesicle proteins were expressed differently between virus-infected and virus-free *C. parasitica* strains [63]. Therefore, the influence of CHV1/EP713 on *C. parasitica* is not only limited to its pathogenesis.

Infection structures, mainly including appressoria and infection cushions (ICs), play crucial role during the infection process for many plant pathogenic fungi with the function of direct penetration of host plants [41]. ICs, also described as “claw-like” structures [64], are observed when plants are inoculated with mycelia of *B. cinerea* or *S. sclerotiorum* [65,66]. Inhibition or deficiency of IC formation lead to severely debilitation of pathogenicity for both two fungi, and wound inoculation could restore the pathogenicity caused by IC deficiency in varying degrees [43,44,45,67,68]. The inhibition of IC formation was probably responsible for the BcMV1-associated hypovirulence in *B. cinerea* [69]. Nevertheless, the vegetative growth of BcMV1-infected strain CanBc-1 was severely impaired. Moreover, wound inoculation was not able to restore the pathogenicity of strain CanBc-1 [69]. Thus, deficiency in formation of ICs is probably not the only factor responsible for BcMV1-associated hypovirulence in *B. cinerea*. Interestingly, similar to fungal strains infected by BcMV1, isolate Z33 also showed significantly decreased formation of ICs. Nonetheless, the vegetative growth of isolate Z33 was normal and comparable to strain HBtom-459; more importantly, wound inoculation could fully recover the virulence of isolate Z33. Thus, inhibition of IC formation may be the major factor responsible for the hypovirulence of isolate Z33. In addition, co-infection by more viruses was accompanied with more phenotypical changes, such as more severe inhibition of IC formation, reduced mycelial growth, and decreased production of conidia and sclerotia. Moreover, wound inoculation was unable to fully recover the virulence of other derivative isolates. These results indicate more cellular pathways may be influenced by the co-infection of BcFV1 and BcEV1, or by the combined effects of the two or three viruses.

To investigate the underling mechanism responsible for the impaired formation of IC, six IC formation-associated genes were selected and tested for their relative transcriptional levels in strain HBtom-459 and isolate Z33 on both PDA and onion bulb scale. All six genes were required for the formation of IC, as their knock-out mutants were unable to form ICs and showed significant reduced virulence [41,42,43,44,45,46]. Consequently, the decreased transcript levels of *Bccaf1* (Figure 9) in isolate Z33 suggest it is related to the reduced formation of IC in isolate Z33. This is consistent with the *Sscaf1* knockout mutant in *S. sclerotiorum*, as wound inoculation could also restore the virulence of *Sscaf1* knockout mutant [44]. Besides participating in the IC formation process of *S. sclerotiorum*, *Sscaf1* may be also involved in sclerotium formation, as the *Sscaf1* knockout mutant produced fewer but larger sclerotia than the wild-type strain [44]. However, isolate Z33 produced more but smaller sclerotia than strain HBtom-459, which is opposite to the case in *S. sclerotiorum*. Some factors may be responsible for the differences, like the transcription of *Bccaf1* was decreased, not null in Z33; other pathways may be affected by viral infection; or the two fungi may have evolved different pathways for sclerotium formation. Therefore, the infection of BcHV1 may affect the expression of certain IC formation-associated genes including *Bccaf1* directly or indirectly, causing the decreased formation of IC, resulting in the hypovirulence of *B. cinerea*. Transcriptome analysis may be helpful to uncover the overall view of gene expressions altered by the infection of BcHV1 in future.

Besides Hubei Province where strain HBtom-372 was originally isolated, BcHV1 was detected in strains from four other provinces, namely Hunan, Jiangxi, Jilin, and Shangdong, occupying a total area of about 907,800 square kilometers for all five provinces (Table S2 and Figure S10). The absence of BcFV1-infected *B. cinerea* strain in Hunan and Jiangxi is most likely due to the small population size (one or two strains) from these two provinces considering their proximity to Hubei province. It is interesting that, in most cases, BcFV1 was detected to be co-infected with BcHV1, as only 1 out of 14 strains showed the BcFV1 infection alone. This is also coincidence with the horizontal transmission of BcHV1 and BcFV1, as most derivative isolates (32 out of 33 isolates) were co-infected by both BcHV1 and BcFV1. Whether BcFV1 has some inner connections with BcHV1 is worth to be tested in further studies. These results indicate that both BcHV1 and BcFV1 have a wide geographic distribution and can spread in natural *B. cinerea* populations.

## Figures and Tables

**Table 1:** Yield of conidium and sclerotium, and size of sclerotium produced by different strains of *Botrytis cinerea.*

Strain	Pathogenicity ^1^	Conidia ^2^	Sclerotium	
		Log_10_ Conidia/Dish (*n* = 5)	Sclerotia/Dish (*n* = 3)	Size (mm) (*n* = 100)
HBtom-459	V	6.95 a ^3^	38 c	3.6 × 2.5
Z33	HV	6.64 b	79 b	2.8 × 2.3
Z1	HV	6.3 c	66 b	2.2 × 1.9
Z3	HV	6.36 b,c	115 a	3.5 × 2.4
Z26	HV	0 d	0 d	NS ^4^
HBtom-372	HV	0 d	0 d	NS

^1^ V = Virulent; HV = Hypovirulent. ^2^ Conidia and sclerotia were collected from 30-day-old cultures grown on potato dextrose agar (20 °C). ^3^ Numbers in the same column followed by the same letters are not significantly different (*p* > 0.05). ^4^ NS = no sclerotial formation.

**Table 2:** Summary of function analysis of six infection cushion formation associated genes in *Botrytis cinerea* or *Sclerotinia sclerotiorum*.

Gene	Description	D ^1^	V ^2^	IC ^3^	CATs ^4^	References
*Bciqg1*	IQGAP homolog	+	+	+	+	[41]
*Bcmsb2*	Sensor protein	-	+	+	unknow	[43]
*Bcpdi1*	ER protein	+	+	+	+	[42]
*Sssac1*	Adenylate cyclase	+	+	+	unknow	[45]
*Ssrgb1*	B regulatory 55-kDa R2 subunit	+	+	+	unknow	[46]
*Sscaf1*	Putative Ca^2+^-binding protein with an EF-hand motif	+	+	+	unknow	[44]

^1^ D: differentiation (includes all tested growth characteristics). ^2^ V: virulence altered in respective deletion mutant (+), not altered (−). ^3^ IC: infection cushions. ^4^ CATs: conidial anastomosis tubes.

## Supplementary Materials

The following are available online at http://www.mdpi.com/1999-4915/10/5/254/s1, Table S1: Oligonucleotide primers/adaptor used for cDNA cloning and revere transcription (RT)-PCR detection of different mycoviruses and dsRNAs in this study. Table S2: The presence of Botrytis cinerea hypovirus 1 (BcHV1) and Botrytis cinerea fusarivirus 1 (BcFV1) in the population of *Botrytis cinerea* isolated from Hubei, Shangdong, Hunan, Jiangxi and Jilin Province, China. Table S3: Oligonucleotide primers/adaptor used for quantitative reverse transcription (qRT)-PCR detection of infection formation related gene in *Botrytis cinerea* strains. Table S4: Sequence identities of Botrytis cinerea hypovirus 1 (BcHV1) to other viruses through multiple alignments of the polyprotein sequences and the amino acid (aa) residue sequences of different domains. Table S5: Sequence identities of Botrytis cinerea fusarivirus 1 (BcFV1) to other viruses through multiple alignments of the polyprotein sequences and the amino acid (aa) residue sequences of different domains. Table S6: Data used to generate histogram. Figure S1: Schematic representation of the strategy used for full cDNA sequence cloning of BcHV1 and BcFV1. PCR primers and the 3′-adaptor used for the cDNA cloning are shown on the diagram. The sequences of the primers and the 3′-adaptor are listed in Table S1. Figure S2: Schematic representation of the strategy used for full cDNA sequence cloning of dsRNA-C, dsRNA-D, dsRNA-E and dsRNA-F. PCR primers and the 3′-adaptor used for the cDNA cloning are shown on the diagram. The sequences of the primers and the 3′-adaptor are listed in Table S1. Figure S3: Pathogenicity assay (**A**); and lesion diameter (**B**) on detached tomato leaves of strains HBtom-372 and HBtom-459 (20 °C, 48 h, lower). “**” indicates a significant difference (*p* < 0.01) between strains HBtom-372 and HBtom-459 in pathogenicity. (**C**) Agarose gel electrophoresis of dsRNAs extracted from the mycelium of *Botrytis cinerea* strains HBtom-372 and HBtom-459. Figure S4: Multiple alignment of amino acid sequences of conserved domains including papain-like protease domain (Prot) and UDP glucose/sterol glucosyltransferase domain (UGT) of BcHV1, and viral RNA Helicase domain (Hel) of both BcHV1 and BcFV1. Motifs in the corresponding domains are indicated by the roman numerals I–VIII. “*” indicates identical amino acid residues; and “.” indicates low chemically similar amino acid residues. Figure S5: Transmembrane domains prediction for ORF1 and ORF2 encoded protein of BcFV1, and the ORF1 encoded protein of RnFV1, SsFV1 and PrRV1. Figure S6: A diagram showing the structure of dsRNA-C, dsRNA-D, dsRNA-E and dsRNA-F and their relationship with BcHV1 and BcFV1. The dashed line rectangle represents the corresponding deletion range on the genome BcHV1, BcFV1 and dsRNA-C. The arrows and sequences in the dashed line frame indicate the extra-nucleotides on dsRNA-D and dsRNA-E in comparison with BcHV1. The gray shades linked both 5′- and 3′-termini of BcHV1 and dsRNA-C indicates the homologous region between BcHV1 and dsRNA-C. Figure S7: Enlarged colony morphology from Figure 6A, red arrowheads indicate the inhibition of mycelial growth of HBtom-459 in duel culture during viral horizontal transmission (20 °C, 7 days). Figure S8: RAPD profiling for different strains of *Botrytis cinerea* using random primer OPC-04. Figure S9: IC formation (enlarged from Figure 8) of different strains/isolates on epidermis of onion bulbs after staining with methyl blue. Figure S10: Geographic distribution of Botrytis cinerea hypovirus 1 (BcHV1) and Botrytis cinerea fusarivirus 1 (BcFV1) in provinces Hubei, Hunan, Jiangxi, Shandong and Jilin, China. The five provinces are highlighted in grey on the map. The numbers of *B. cinerea* strains are labeled as “number of total strains/number of BcHV1 infected strains/number of BcFV1 infected strains” in the parentheses for each province.
