# Supplementary material for: Two Novel Hypovirulence-Associated Mycoviruses in the Phytopathogenic Fungus Botrytis cinerea: Molecular Characterization and Suppression of Infection Cushion Formation
Source: Viruses. 2018 May 13;10(5):254. doi: 10.3390/v10050254 (PMC5977247; doi:10.3390/v10050254)
Supplement: Supplementary file 1 [file viruses-10-00254-s001.pdf]

Table S1 Oligonucleotide primers/adaptor used for cDNA cloning and revere transcription (RT)-PCR detection of different mycoviruses and dsRNAs in this study

Primer Name	Sequence (5′→ 3′)	Position[1]	Polarity[2]
Full cDNA cloning			
HV-R (BcHV1)	CCAGGCAGGAAACAAGC	1680-1697	–
HV0-R (BcHV1)	CCCAATAGCGAAACCAAC	2723-2741	–
HV1-R (BcHV1)	TCAAAGGATGGTGCAAGG	4521-4539	–
HV2-F (BcHV1)	ACGCTGTCCAACGTATAGAA	5139-5159	+
HV2-R (BcHV1)	CACCAGATTGAGCCCTTT	7950-7968	–
HV3-F (BcHV1)	TTAGCAAGGGAAGGAACT	8412-8430	+
HV4-F (BcHV1)	CCTGGAGGCTACGAAGAT	8966-8984	+
FV1-R (BcFV1)	GCAGGAGTTGCGATAAGG	1210-1228	–
FV2-F (BcFV1)	ACCCTCAAGTGAAGAAAGC	5653-5672	+
FV3-F (BcFV1)	ACTGTGGAGGAAGAAGAGC	6655-6674	+
CV1-R (dsRNA-C)	GGCAGAGGAGGGTTGATG	1369-1386	–
CV2-F (dsRNA-C)	CGTTTGAGGCTTCTACCA	2886-2903	+
DV1-R(dsRNA-D)	GCGGACCACGTCATTCAA	1904-1921	–
DV2-F(dsRNA-D)	ACTCACCGTTGCCCTCAA	2798-2815	+
EV1-R(dsRNA-E)	AACATTGAAGCGACTGGA	1927-1946	–
EV2-F(dsRNA-E)	AACCCAGAGGCATACGAG	2782-2799	+
110A (3′-adaptor)	TATCTTATCGGCGTGTCCCCC	to 3′-end of dsRNA	+/–
RC110A (primer)	GGGGGACACGCCGATAAGATA	complementary to the adaptor 110A	–/+
RT-PCR detection of different mycoviruses and dsRNAs			
H-RT-F (BcHV1)	ACCGATTGGTTGAAGTGA	5514-5532	+
H-RT-R (BcHV1)	TTGCCATAGAAAGGTAGTAGTT	6212-6234	–
F-RT-F (BcFV1)	AGCAAGATGGCAAGAAAA	2586-2604	+

F-RT-R (BcFV1)	ACGTGCAGCACTAGCAGA	3318-3336	–
C-RT-F (dsRNA-C)	TTCGGGTGGCGATGT	1024-1038	+
C-RT-R (dsRNA-C)	TTGGCACTAAGGAAGTTATGT	1728-1748	–
RT-F-F (dsRNA-F)	CCTTATTGCAAGGCCAGCATGTCA	136-159	+
RT-F-R (dsRNA-F)	TGAACCTTTCTCGAGCATTGCTCAT	783-807	–
to probe BcHV1 and BcFV1			
Probe 1-F (BcHV1)	ACCGATTGGTTGAAGTGA	5514-5532	+
Probe 1-R (BcHV1)	TTGCCATAGAAAGGTAGTAGTT	6212-6234	–
Probe 2-F (BcFV1)	AGCAAGATGGCAAGAAAA	2586-2604	+
Probe 2-R (BcFV1)	ACGTGCAGCACTAGCAGA	3318-3336	–
RAPD primer			
OPC-04	CCGCATCTAC		

[1] Positions for the PCR primers or the adaptor in the cDNA of BcHV1 and BcFV1 were labeled in Fig. S1.

[2] Polarity refers to the positive strand (+) and the negative strand (–) of BcHV1 and BcFV1.

Table S2 The presence of Botrytis cinerea hypovirus 1 (BcHV1) and Botrytis cinerea fusarivirus 1 (BcFV1) in the population of *Botrytis cinerea* isolated from Hubei, Shandong, Hunan, Jiangxi and Jilin Province, China.

Location	Number of Strains (host, presence of mycoviruses)		
	Total	BcHV1+[1]	BcFV1+
Wuhan, Hubei	5	1	0
Yichang, Hubei	7	1	1[2]
Suizhou, Hubei	5	0	0
Xiantao, Hubei	7	0	0
Qianjiang, Hubei	6	0	0
Xiaogan, Hubei	8	2	1
Xiangyang, Hubei	6	3	0
Jingmen, Hubei	5	3	3
Huangshi, Hubei	4	1	0
Shiyan, Hubei	3	1	0
Jingzhou, Hubei	7	1	0
Huanggang, Hubei	6	3	0
Enshi, Hubei	3	0	0
Xianning, Hubei	6	0	0
Ezhou, Hubei	6	0	0
Weifang, Shandong	7	7	5
Zibo, Shandong	1	1	1

Changsha, Hunan	2	1	0
Jiujiang, Jiangxi	1	1	0
Changchun, Jilin	2	2	2
Siping, Jilin	1	1	1
Total	98	29	14

[1] "+" represents the presence of BcHV1 or BcFV1 through the detection of RT-PCR with primer pairs listed in Table S1.

[2] The strain of *B. cinerea* infected by BcFV1 is not the same strain infected by BcHV1 in Yichang, Hubei.

Table S3 Oligonucleotide primers/adaptor used for quantitative reverse transcription (qRT)-PCR detection of infection formation related gene in *Botrytis cinerea* strains

Gene	Primer Name	Sequence (5′→ 3′)	Accession no.	References[1]
Bciqg1	Bciqg1-F	TTGCCCGGAACCTTTGTCAT	XM_001555933	(Marschall and Tudzynski, 2016)
	Bciqg1-R	GCAAGAACCTTTGGCACGTT		
Bcmsb2	Bcmsb2-F	CTTGGATCAGCCACTTCGGT	XM_001550277	(Leroch et al., 2015)
	Bcmsb2-R	GTAGCCGAGGCAGATACACC		
Bcpdi1	Bcpdi1-F	CGATATCCTCGCTGGCCTTT	XM_001559009	(Marschall and Tudzynski, 2017)
	Bcpdi1-R	GCTACGGCTACCGTTGTAGG		
Bcsac1	Bcsac1-F	TGCCACTCTTGCTCCTGATTC	AJ276473	(Jurick and Rollins, 2007)
	Bcsac1-R	ACCTCCCCACCTCTGTTTTTC		
Bcrgb1	Bcrgb1-F	AAAAATCATAGACCGTTTCCGC	XM_001546540	(Erental et al., 2007)
	Bcrgb1-R	CATCGTAGAGAACCTGGTAGCC		
Bccaf1	Bccaf1-F	CGGGCTCTTGGCTTGTGT	XM_001556300	(Xiao et al., 2014)
	Bccaf1-R	TCCGCTTGGTCCCATACG		
Bcactin	Bcact-F	TCCAAACCGCCAGTCAATCC	AJ000335	（Liu *et al*., 2011）
	Bcact-R	GATACCACCGCTCTCAAGACC		

[1] Xiao, X.Q.; Xie, J.T.; Cheng, J.C.; Li, G.Q.; Yi, X.H.; Jiang, D.H.; Fu, Y.P. Novel Secretory Protein Ss-Caf1 of the Plant-Pathogenic Fungus *Sclerotinia sclerotiorum* Is Required for Host Penetration and Normal Sclerotial Development. Mol Plant Microbe Interact 2014, 27, 40–55.

Leroch, M.; Mueller, N.; Hinsenkamp, I.; Hand, M. The signalling mucin Msb2 regulates surface sensing and host penetration via BMP1 MAP kinase signalling in *Botrytis cinerea.* Molecular plant pathology 2015, 16, 787–798

Marschall, R.; Tudzynski, P. BcIqg1, a fungal IQGAP homolog, interacts with NADPH oxidase, MAP kinase and

calcium signaling proteins and regulates virulence and development in *Botrytis cinerea*. Mol Microbiol 2016, 101, 281–298.

Marschall, R.; Tudzynski, P. The Protein Disulfide Isomerase of *Botrytis cinerea*: An ER Protein Involved in Protein Folding and Redox Homeostasis Influences NADPH Oxidase Signaling Processes. Front Microbiol 2017, 8, 1–15.

Jurick, W.M.; Rollins, J.A. Deletion of the adenylatecyclase (*sac1*) gene affects multiple developmental pathways and pathogenicity in *Sclerotinia sclerotiorum*. Fungal Genet Biol 2007, 44, 521–530.

Erental, A.; Harel, A.; Yarden, O. Type 2A phosphoprotein phosphatase is required for asexual development and pathogenesis of *Sclerotinia sclerotiorum*. Mol Plant Microbe Interact 2007, 20, 944–954.

Liu, W.; Soulié, M.C.; Perrino, C.; Fillinger, S. The osmosensing signal transduction pathway from *Botrytis cinerea* regulates cell wall integrity and MAP kinase pathways control melanin biosynthesis with influence of light. *Fungal Genet Biol* 2011, 48, 377–387

Table S4. Sequence identities of Botrytis cinerea hypovirus 1 (BcHV1) to other viruses through multiple alignments of the polyprotein sequences and the amino acid (aa) residue sequences of different domains

Family	Virus	Acronym	aa identity					Accession no.
			Full	Prot	UGT	RdRp	Hel	
Hypoviridae	Sclerotinia sclerotiorum hypovirus 1	SsHV1	67.4	88.57	63.34	85.94	71.48	NC_015939.1
	Cryphonectria hypovirus 3	CHV3	58.31	26.02	69.7	82.03	40.74	NC_000960.1
	Phomopsis longicolla hypovirus	PlHV1	57.84	11.32	67.44	85.16	69.63	NC_024685.1
	Valsa ceratosperma hypovirus 1	VcHV1	56.48	32.46	69.37	78.13	66.30	NC_017099.1
	Cryphonectria hypovirus 4	CHV4	44.55	27.05	57.61	75.78	54.24	NC_006431.1
	Cryphonectria hypovirus 1	CHV1	5.43	11.43	-	11.03	21.60	NC_001492.1
	Cryphonectria hypovirus 2	CHV2	6.14	10.28	-	10.31	21.33	NC_003534.1
	Fusarium graminearum hypovirus 1	FgHV1	4.86	12.26	-	12.1	19.51	NC_023680.1
	Fusarium graminearum hypovirus 2	FgHV2	4.36	9.43	-	11.22	20	NC_026813.1
	Macrophomina phaseolina hypovirus	MpHV1	3.90	-	-	15.38	22.34	KP900893.1
	Sclerotinia sclerotiorum hypovirus 2	SsHV2	3.55	13.89	-	15.08	14.42	KJ561218.1
Fusariviridae	Sclerotinia sclerotiorum fusarivirus 1	SsFV1	8.83	-	-	29.39	18.61	NC_027208.1
	Penicillium roqueforti ssRNA	PrRV1	8.18	-	-	26.74	20.34	NC_024699.1
	Rosellinia necatrix fusarivirus 1	RnFV1	8.58	-	-	26.36	21.02	NC_024485.1
	Fusarium graminearum dsRNA	FgV1	8.33	-	-	24.62	21.45	NC_006937
	Fusarium poae fusarivirus 1	FpFV1	7.9	-	-	20.59	19.86	NC_030868.1
	Pleospora typhicola fusarivirus 1	PtFV1	8.7	-	-	21.97	19.73	NC_028470.1
	Penicillium aurantiogriseum	PaFV1	8.3	-	-	22.95	19.79	NC_028467.1
	Alternaria brassicicola fusarivirus 1	AbFV1	8.91	-	-	21.73	20.61	NC_029056.1
	Nigrospora oryzae fusarivirus 1	NoFV1	8.09	-	-	21.78	21.43	KU980909.1
	Macrophomina phaseolina	MpSRV1	8.17	-	-	28.85	21.18	KP900890.1

[1] The full sequence means the larger ORF for alphahypoviruses and fusariviruses which contain the RdRp or Hel domain.

Table S5 Sequence identities of Botrytis cinerea fusarivirus 1 (BcFV1) to other viruses through multiple alignments of the polyprotein sequences and the amino acid (aa) residue sequences of different domains

Family	Virus	Acronym	aa identity			Accession no.
			Full sequence[1]	RdRp	Hel	
Fusariviridae	Alternaria brassicicola fusarivirus 1	AbFV1	25.58	47.17	32.44	NC_029056.1
	Sclerotinia sclerotiorum fusarivirus 1	SsFV1	24.30	46.98	33.22	NC_027208.1
	Pleospora typhicola fusarivirus 1	PtFV1	23.84	44.59	29.61	NC_028470.1
	Fusarium poae fusarivirus 1	FpFV1	23.83	44.13	29.63	NC_030868.1
	Fusarium graminearum dsRNA mycovirus-1	FgV1	23.72	46.31	28.96	NC_006937
	Penicillium roqueforti ssRNA mycovirus 1	PrRV1	23.51	43.63	27.61	NC_024699.1
	Penicillium aurantiogriseum fusarivirus 1	PaFV1	22.77	38.85	30.43	NC_028467.1
	Rosellinia necatrix fusarivirus 1	RnFV1	22.73	40.95	27.09	NC_024485.1
	Nigrospora oryzae fusarivirus 1	NoFV1	22.55	37.76	31.94	KU980909.1
	Macrophomina phaseolina single-stranded RNA virus 1	MpSRV1	22.26	38.54	27.85	KP900890.1
Hypoviridae	Cryphonectria hypovirus 3	CHV3	7.35	25.8	23.57	NC_000960.1
	Cryphonectria hypovirus 4	CHV4	7.29	27.39	22.37	NC_006431.1

	Sclerotinia sclerotiorum hypovirus 1	SsHV1	7.10	28.8	23.91	NC_015939.1
	Valsa ceratosperma hypovirus 1	VcHV1	7.46	27.07	22.90	NC_017099.1
	Phomopsis longicolla hypovirus	PlHV1	7.77	26.43	23.75	NC_024685.1
	Cryphonectria hypovirus 1	CHV1	6.73	14.07	12.62	NC_001492.1
	Cryphonectria hypovirus 2	CHV2	6.1	14.14	14.10	NP_613266
	Fusarium graminearum hypovirus 1	FgHV1	5.34	16.01	15.91	NC_023680.1
	Fusarium graminearum hypovirus 2	FgHV2	5.47	13.49	17.18	NC_026813.1
	Macrophomina phaseolina hypovirus 1	MpHV1	4.79	14.36	16.83	KP900893.1
	Sclerotinia sclerotiorum hypovirus 2	SsHV2	4.74	12.62	14.06	KJ561218.1
Potyviridae	Barley mild mosaic virus	BmMV	9.86	22.33	16.17	AJ242725.1
	Wheat yellow mosaic virus	WyMV	4.43	20.19	-	AB627812.1
Poxviridae	Pseudocowpox virus	PCPV	6.68	-	19.88	GQ329669.1
	Bovine papular stomatitis virus	BPSV	7.29	-	20.43	NC_005337.1
Iflaviridae	Opsiphanes invirae iflavirus 1	Oily-1	5.59	18.04	-	KR534892.1
Secoviridae	Strawberry mottle virus	SMoV	6.60	17.39	-	NP_599086.1

[1] The full sequence means the larger ORF for alphahypoviruses and fusariviruses which contain the RdRp or Hel domain.

Table S6. Data used to generate histogram

Figure 1

Strain	Lesion diameter (mm)	Growth rate (mm/d)
HBtom-372	0.55 ± 1.79	2.03 ± 0.78
B05.10	18 ± 1.5	15.08 ± 0.38

Figure 7

Strain	Growth rate (mm/d)	Lesion diameter (mm) Wounded
HBtom-372	2.63 ± 0.87 d	1.3 ± 2.91 c
Z26	4.38 ± 1.25 c	7.1 ± 1.78 b
Z1	4.81 ± 1.65 c	1.1 ± 1.75 c
Z3	9.38 ±1.51 b	1 ± 2.24 c
Z33	15.75 ± 0.96 a	4 ± 3.76 bc
HBtom-459	14.56 ± 2.08 a	19.7 ± 1.51 a

Figure 8

Strain	Lesion diameter (mm) Intact	Lesion diameter (mm) Wounded	Number of infection cushion
HBtom-372	0.6 ± 1.87 efg[1]	2.85 ± 4.06 de	0 ± 0 d
Z26	4.65 ± 3.54 d	10.45 ± 0.99 c	14.67 ± 1.15 cd
Z1	0.35 ±1 .56 fg	2.55 ± 3.66 def	10.67 ± 5.03 cd
Z3	0 ± 0 g	0.7 ± 2.17 efg	20 ± 4 c
Z33	3.75 ± 5.39 d	17.35 ± 1.84 ab	55.33 ± 3.06 b
HBtom-459	16.45 ± 1.27 b	19.35 ± 2.25 a	195.33 ± 24.44 a

Figure S1

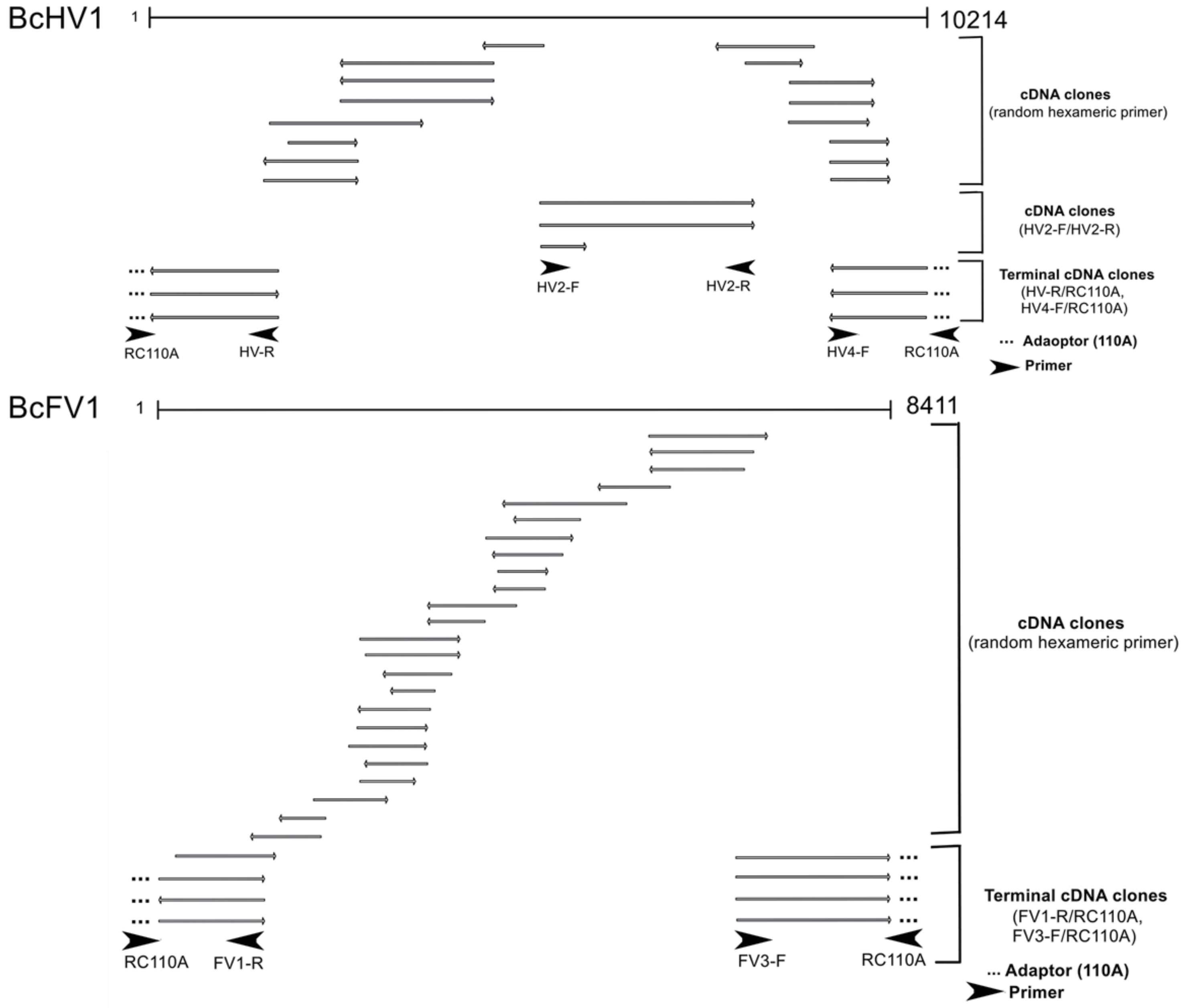
BcHV1
1
10214
cDNA clones
(random hexameric primer)
cDNA clones
(HV2-F/HV2-R)
Terminal cDNA clones
(HV-R/RC110A,
HV4-F/RC110A)
HV2-F
HV2-R
RC110A
HV-R
HV4-F
RC110A
... Adaoptor (110A)
Primer
BcFV1
1
8411
cDNA clones
(random hexameric primer)
Terminal cDNA clones
(FV1-R/RC110A,
FV3-F/RC110A)
RC110A
FV1-R
FV3-F
RC110A
... Adaptor (110A)
Primer

Figure S2

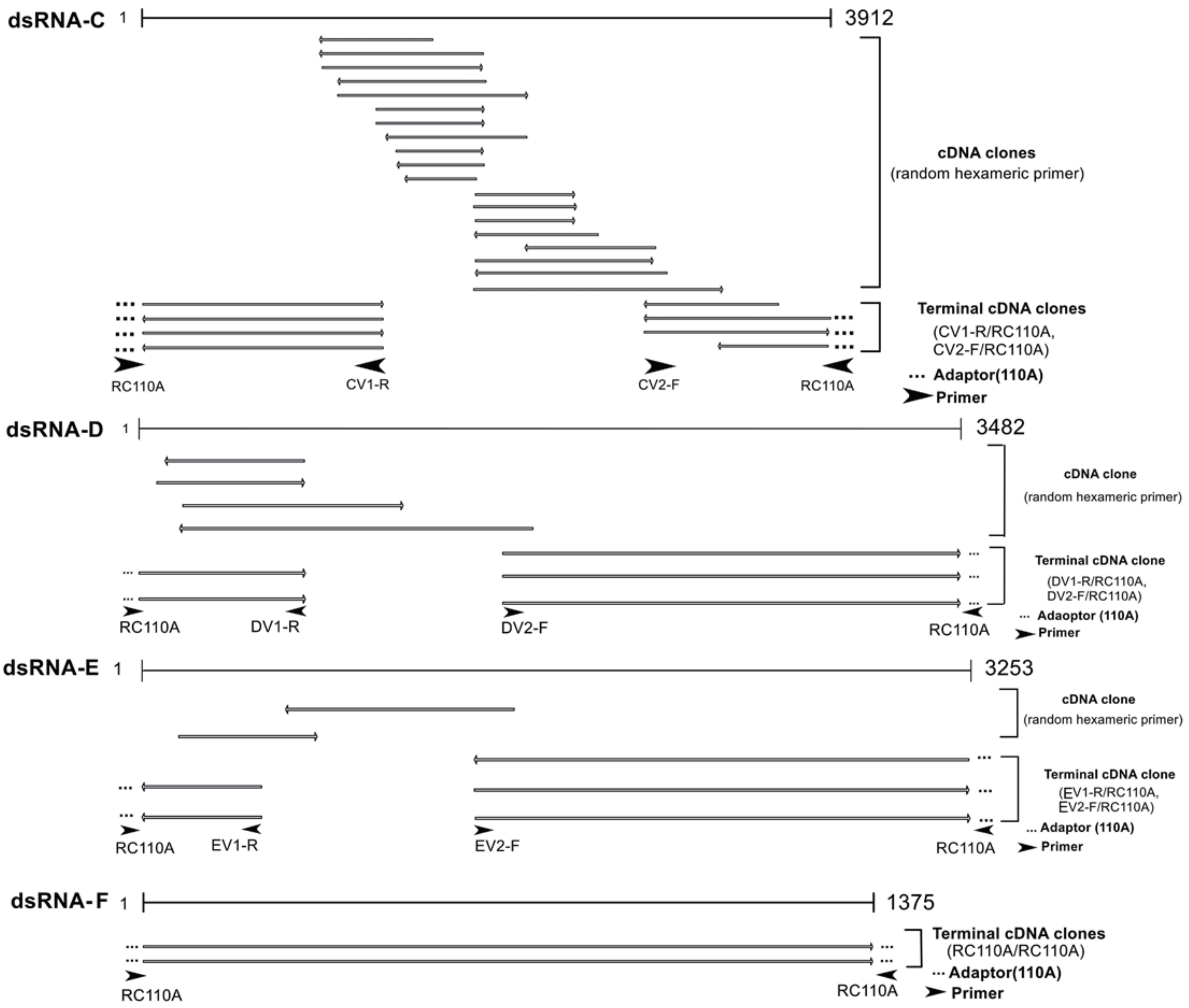

dsRNA-C
1
3912
cDNA clones
(random hexameric primer)
Terminal cDNA clones
(CV1-R/RC110A,
CV2-F/RC110A)
RC110A
CV1-R
CV2-F
RC110A
Adaptor(110A)
Primer
dsRNA-D
1
3482
cDNA clone
(random hexameric primer)
Terminal cDNA clone
(DV1-R/RC110A,
DV2-F/RC110A)
RC110A
DV1-R
DV2-F
RC110A
Adaoptor (110A)
Primer
dsRNA-E
1
3253
cDNA clone
(random hexameric primer)
Terminal cDNA clone
(EV1-R/RC110A,
EV2-F/RC110A)
RC110A
EV1-R
EV2-F
RC110A
Adaptor (110A)
Primer
dsRNA-F
1
1375
Terminal cDNA clones
(RC110A/RC110A)
RC110A
RC110A
Adaptor(110A)
Primer

Figure S3

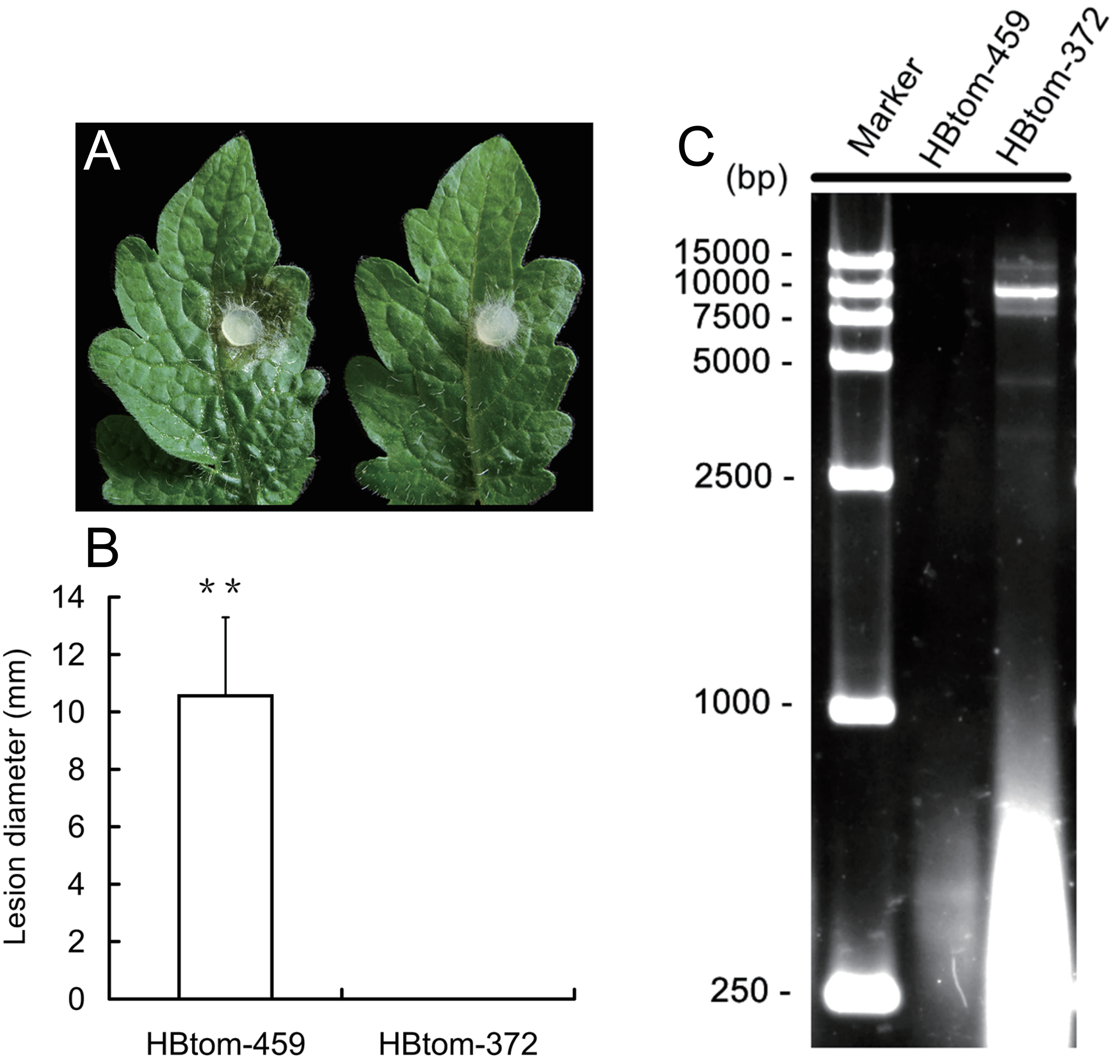

A
B
C
Marker
HBtom-459
HBtom-372
(bp)
15000 -
10000 -
7500 -
5000 -
2500 -
1000 -
250 -
Lesion diameter (mm)
14
12
10
8
6
4
2
0
* *
HBtom-459
HBtom-372

Figure S4

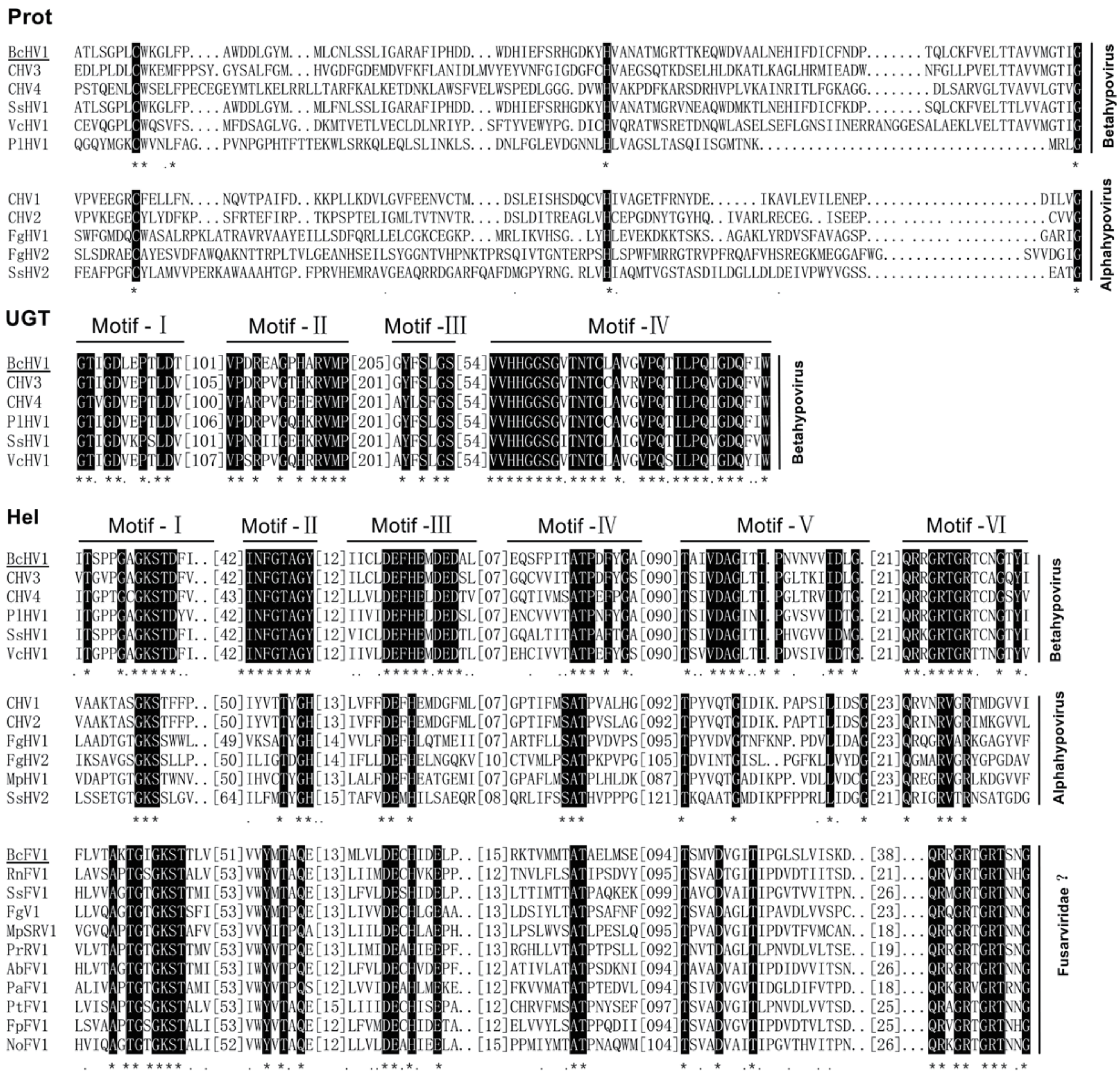

Prot
UGT
Hel
Motif - I
Motif - II
Motif - III
Motif - IV
Motif - V
Motif - VI
Betahypovirus
Alphahypovirus
Fusarviridae ?

Figure S5

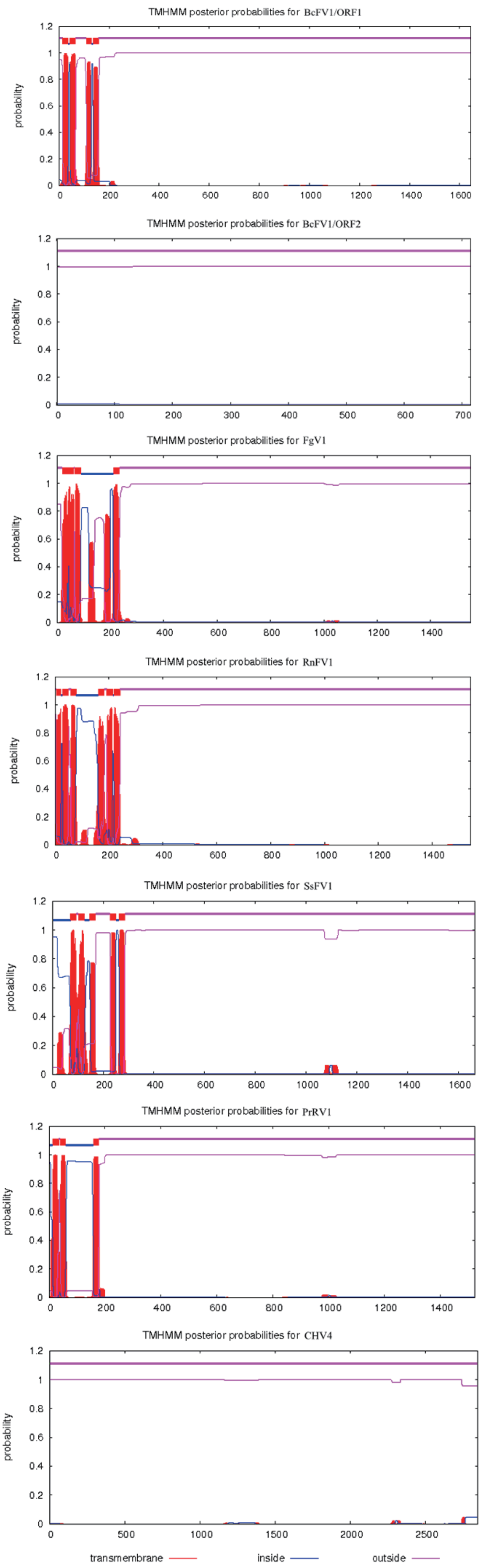
TMHMM posterior probabilities for BcFV1/ORF1
probability
1.2 1 0.8 0.6 0.4 0.2 0
0 200 400 600 800 1000 1200 1400 1600
TMHMM posterior probabilities for BcFV1/ORF2
probability
1.2 1 0.8 0.6 0.4 0.2 0
0 100 200 300 400 500 600 700
TMHMM posterior probabilities for FgV1
probability
1.2 1 0.8 0.6 0.4 0.2 0
0 200 400 600 800 1000 1200 1400
TMHMM posterior probabilities for RnFV1
probability
1.2 1 0.8 0.6 0.4 0.2 0
0 200 400 600 800 1000 1200 1400
TMHMM posterior probabilities for SsFV1
probability
1.2 1 0.8 0.6 0.4 0.2 0
0 200 400 600 800 1000 1200 1400 1600
TMHMM posterior probabilities for PrRV1
probability
1.2 1 0.8 0.6 0.4 0.2 0
0 200 400 600 800 1000 1200 1400
TMHMM posterior probabilities for CHV4
probability
1.2 1 0.8 0.6 0.4 0.2 0
0 500 1000 1500 2000 2500
transmembrane
inside
outside

Figure S6

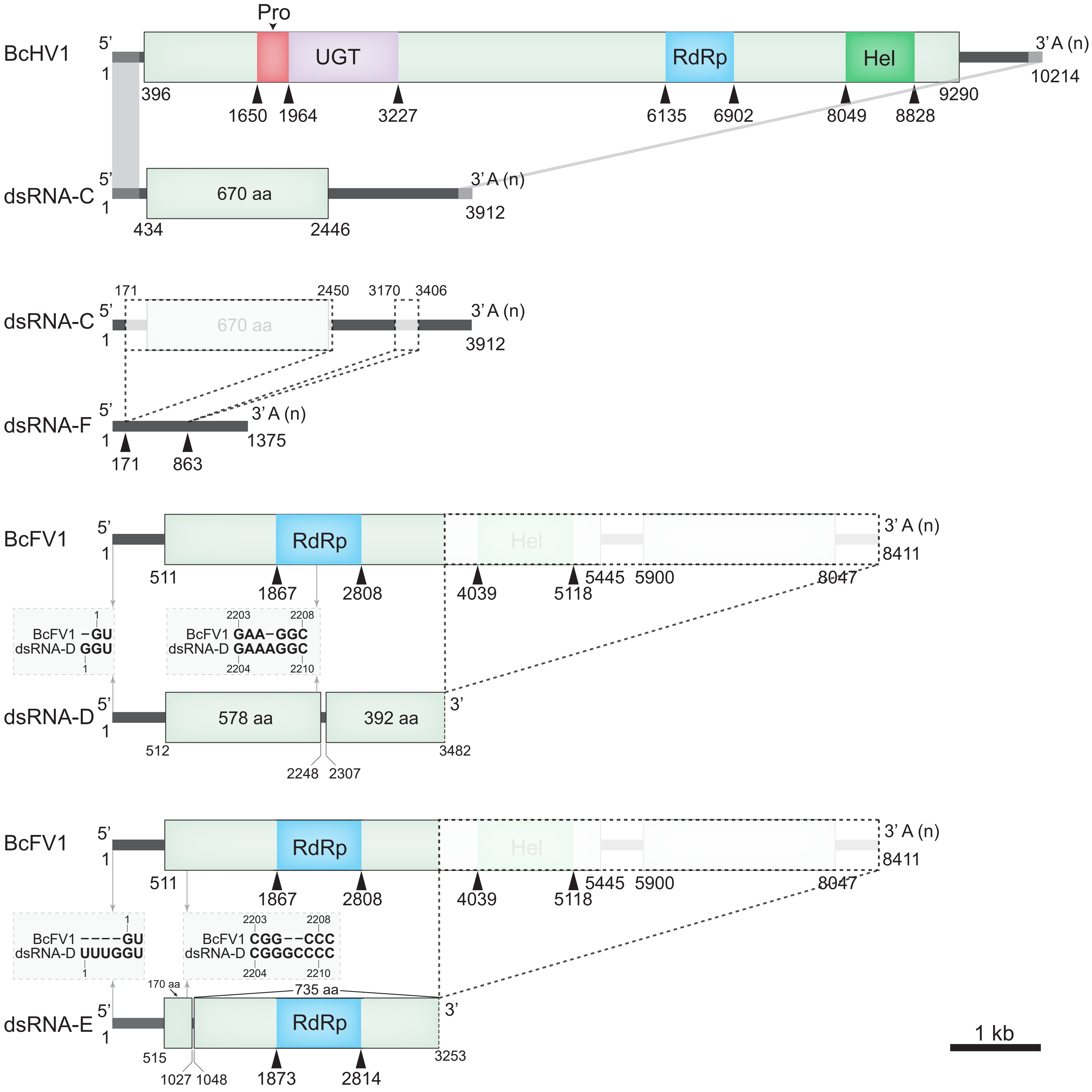
BcHV1
Pro
UGT
RdRp
Hel
5'
1
3' A (n)
10214
396
1650
1964
3227
6135
6902
8049
8828
9290
dsRNA-C
670 aa
434
2446
3912
171
2450
3170
3406
dsRNA-F
1375
863
BcFV1
8411
511
1867
2808
4039
5118
5445
5900
8047
BcFV1 −GU
dsRNA-D GGU
BcFV1 GAA−GGC
dsRNA-D GAAAGGC
2203
2208
2204
2210
dsRNA-D
578 aa
392 aa
512
2248
2307
3482
3'
BcFV1 −−−−GU
dsRNA-D UUUGGU
BcFV1 CGG−−CCC
dsRNA-D CGGGCCCC
170 aa
735 aa
dsRNA-E
515
1027
1048
1873
2814
3253
1 kb

Figure S7

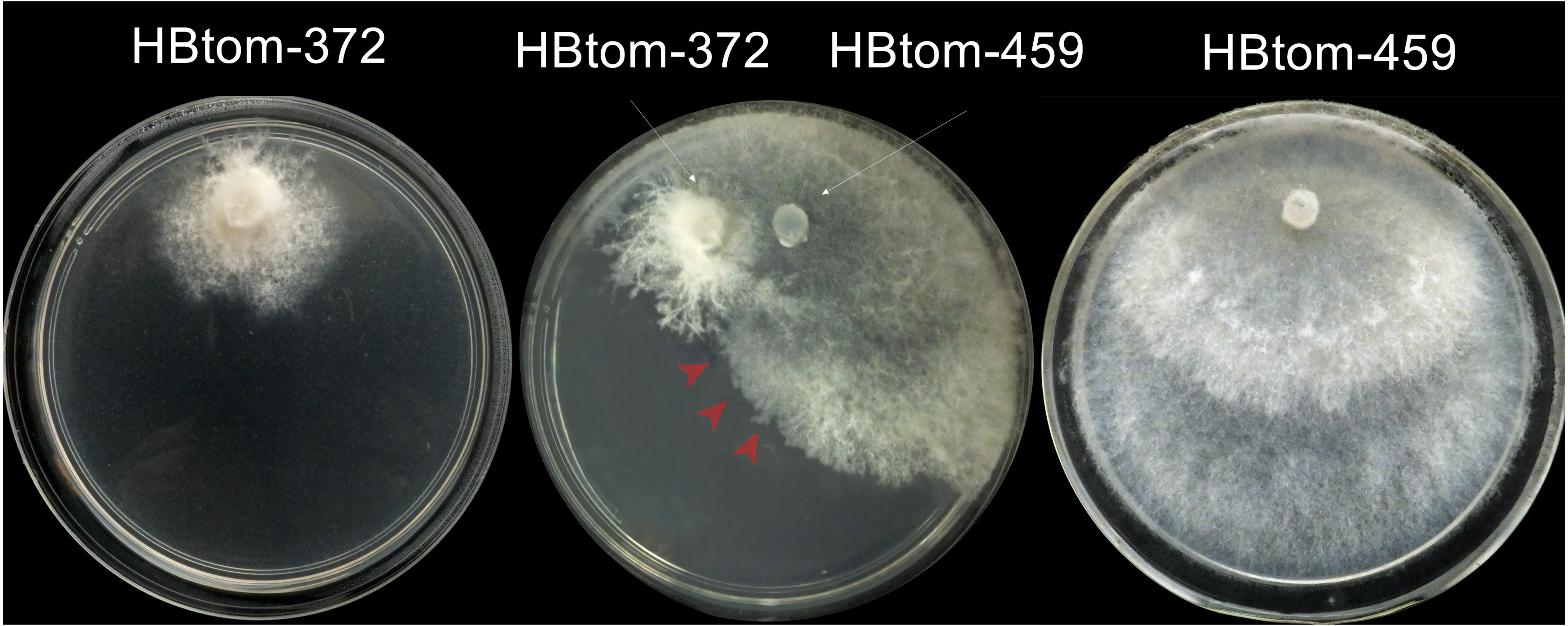

Figure S8

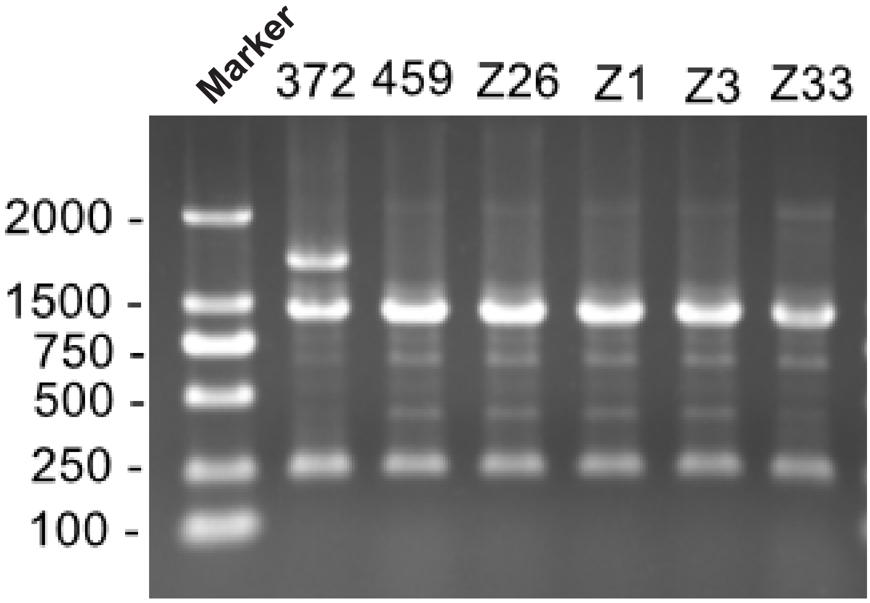

Figure S9

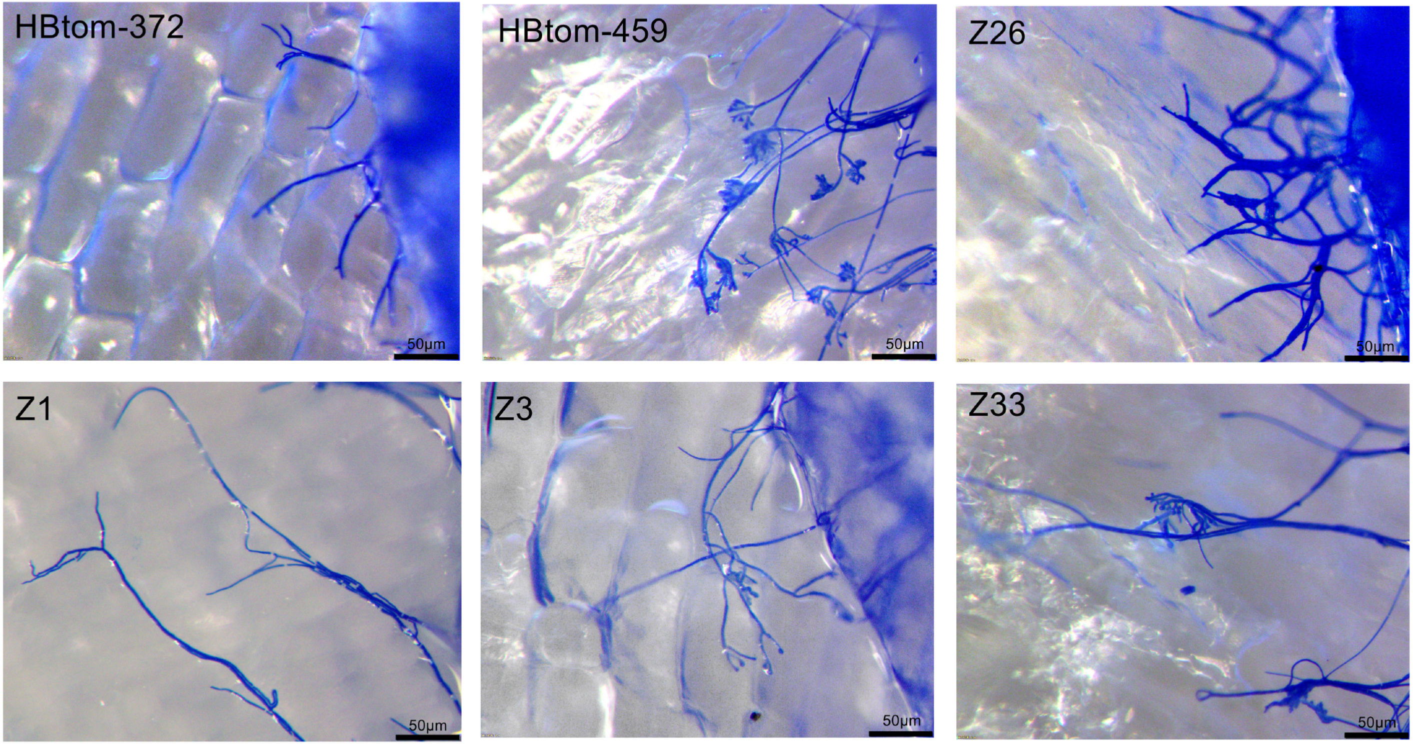
HBtom-372
50μm
HBtom-459
50μm
Z26
50μm
Z1
50μm
Z3
50μm
Z33
50μm

Figure S10

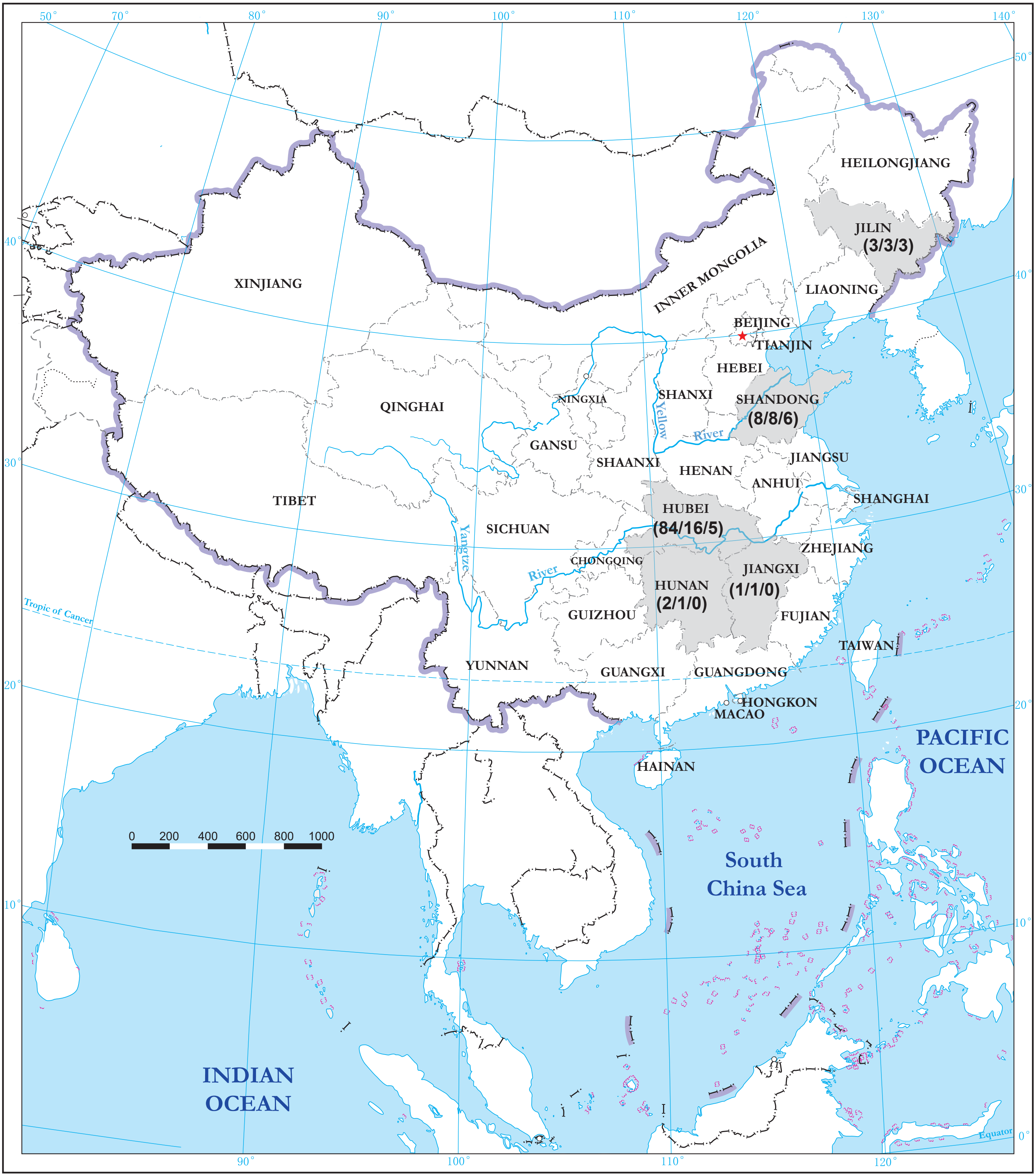

HEILONGJIANG
JILIN
(3/3/3)
LIAONING
INNER MONGOLIA
XINJIANG
BEIJING
TIANJIN
HEBEI
SHANXI
SHANDONG
(8/8/6)
NINGXIA
QINGHAI
GANSU
SHAANXI
HENAN
JIANGSU
ANHUI
SHANGHAI
TIBET
HUBEI
(84/16/5)
SICHUAN
ZHEJIANG
CHONGQING
JIANGXI
(1/1/0)
HUNAN
(2/1/0)
GUIZHOU
FUJIAN
TAIWAN
YUNNAN
GUANGXI
GUANGDONG
HONGKON
MACAO
HAINAN
Yellow River
Yangtze River
Tropic of Cancer
Equator
PACIFIC OCEAN
South China Sea
INDIAN OCEAN
0 200 400 600 800 1000
